# Exercise-associated microbial metabolites prevent skeletal muscle atrophy in adult female mice

**DOI:** 10.1038/s41467-026-74852-w

**Published:** 2026-07-10

**Authors:** Benjamin I. Burke, Taylor R. Valentino, Ahmed Ismaeel, Salim S. El-Amouri, Jensen Goh, Logan N. Scott, Bonnie J. Walton, Jai K. Joshi, Cecily R. Wood, Abigail Burrows-Franco, John B. May, Lance A. Johnson, Michael D. Flythe, Yuan Wen, John J. McCarthy

**Affiliations:** 1 Department of Physiology, College of Medicine, University of Kentucky, Lexington, KY USA; 2 Center for Muscle Biology, College of Health Sciences, University of Kentucky, Lexington, KY USA; 3 Buck Institute for Research on Aging, Novato, CA USA; 4 Department of Anatomy, Physiology and Pharmacology, College of Veterinary Medicine, Auburn University, Auburn, AL USA; 5 Division of Biomedical Informatics, Department of Internal Medicine, College of Medicine, University of Kentucky, Lexington, KY USA; 6 Mass Spectrometry and Proteomics Core, University of Kentucky, Lexington, KY USA; 7 Department of Plant and Soil Sciences, College of Agriculture, Food and Environment, University of Kentucky, Lexington, KY USA; 8 Sanders Brown Center on Aging, University of Kentucky, Lexington, KY USA; 9 USDA Agriculture Research Service, Forage-Animal Production Research Unit, University of Kentucky, Lexington, KY USA; 10 Department of Animal and Food Sciences, College of Agriculture, Food and Environment, University of Kentucky, Lexington, KY USA

**Keywords:** Metabolism, Physiology, Preclinical research, Applied microbiology

## Abstract

We previously reported that skeletal muscle adaptation to regular exercise requires a healthy gut microbiome, contributing to growing evidence that some exercise benefits are mediated by microbiome-derived metabolites. Here, to identify such exercise-associated microbial metabolites, we transfer cecal contents from exercise-trained female donor mice into exercise-naïve female recipient mice undergoing unilateral hindlimb immobilization. Recipients of cecal material from exercise-trained donors exhibit less muscle atrophy compared with those receiving transfers from sedentary donors. Untargeted metabolomics reveal metabolites enriched in cecal content, serum, and muscle of recipients from exercise-trained donors, consistent with microbial origin. Oral administration of two such metabolites (pipecolic acid and succinate) attenuates muscle atrophy and preserves muscle function in exercise-naïve mice, potentially by enhancing cellular energy status and translational capacity. These findings further define the gut microbiome-skeletal muscle axis and provide evidence that exercise-associated microbial metabolites serve as a novel class of exercise mimetics for treating conditions responsive to physical activity.

## Abstract (web summary)

The gut microbiome from exercise-trained mice protects against muscle atrophy when transferred to immobilized mice. Two microbial metabolites, pipecolic acid and succinate, are identified as exercise mimetics that preserve muscle mass and function.

## Introduction

The gut microbiome is comprised of the trillions of microorganisms found within the gastrointestinal tract and functions through a dynamic symbiotic relationship to directly impact the health and well-being of the host. Dysregulation of this complex ecosystem can significantly impair host health^1^. Recent evidence has established a clear interaction between the gut microbiome and skeletal muscle through microbial-derived metabolites^2–5^. We reported that dysbiosis of the gut microbiome impaired skeletal muscle adaptation to exercise training^6^. Further, we and others have shown the direct administration of microbial-derived metabolites has beneficial effects on skeletal muscle^5,7–9^, underscoring the gut microbiome’s importance in muscle adaptation.

The purpose of this study was to test the hypothesis that, in response to exercise training, the gut microbiome produces metabolites which can be used to treat skeletal muscle atrophy. The study was motivated by our finding that a healthy gut microbiome is necessary for skeletal muscle adaptation to exercise training^6^, presumably through the production of metabolites^7^. To test our hypothesis, we first transferred cecal content from sedentary donors (DON-SED) or exercise-trained donors (DON-EXR) into exercise-naïve recipient mice (REC-SED and REC-EXR, respectively) that were undergoing muscle atrophy induced by unilateral hindlimb immobilization (HLI). Upon finding skeletal muscle disuse atrophy was significantly less in REC-EXR group compared to RED-SED group, we used untargeted metabolomics to identify candidate microbial-derived metabolites. The administration of two such metabolites, pipecolic acid and succinate, during unilateral HLI prevented muscle atrophy while preserving muscle function. The effectiveness of these metabolites to maintain muscle mass and function during a period of disuse demonstrates their therapeutic potential and establishes a novel class of exercise mimetics. Given the deep richness of the gut microbiome, this work is presented with the expectation that many more exercise-associated microbial metabolites capable of treating exercise-responsive conditions remain to be discovered.

## Results

### Cecal transfers from exercised donors increase microbial diversity

**Fig. 1:**
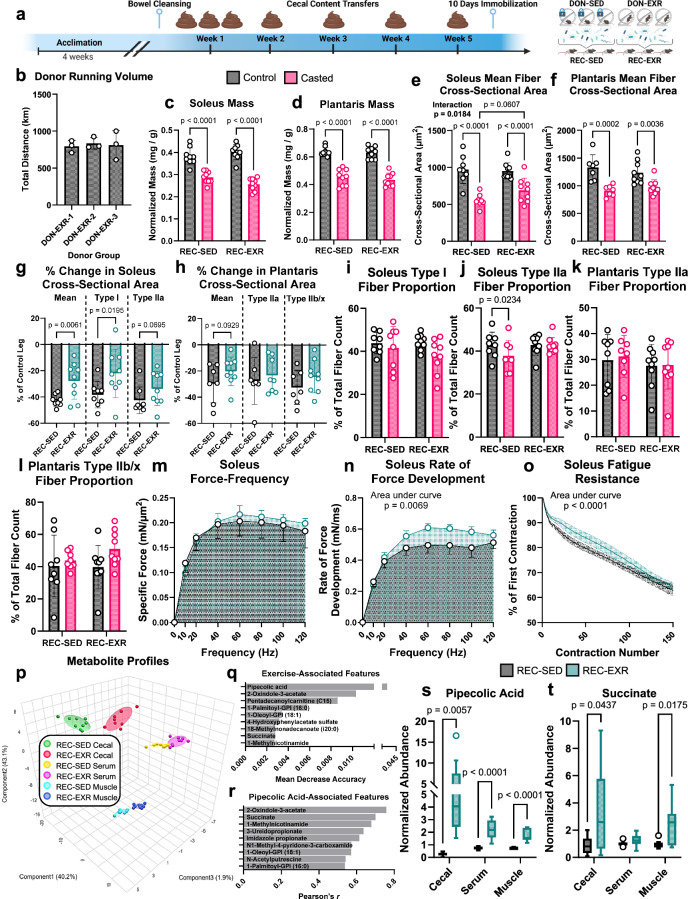
Cecal transfers from exercise-trained donors ameliorate loss in muscle size and function during disuse. **a** Experimental design showing acclimatization period, timeline of cecal transfers from sedentary donors (DON-SED) and exercised donors (DON-EXR) into recipient mice (REC-SED, REC-EXR, respectively), and 10-day hindlimb immobilization; created with BioRender.com. **b** Average running volumes of DON-EXR groups used for cecal transfers; one-way ANOVA with Tukey’s post hoc. **c**,**d** Muscle mass of soleus and plantaris in control and casted legs normalized to body weight of REC-SED and REC-EXR after 10 days of hindlimb immobilization; two-way ANOVA with Fisher’s post hoc. **e**,**f** Mean muscle fiber cross-sectional area (CSA) of soleus and plantaris muscles; two-way ANOVA with Fisher’s post hoc. **g**,**h** Percent change in muscle mean and fiber-type specific CSA in soleus and plantaris muscles; independent one-tailed t-test. **i–l** Muscle fiber type proportions in soleus and plantaris muscles; two-way ANOVA with Fisher’s post hoc. **m** Ex vivo soleus force-frequency relationship; independent one-tailed t-test, *n* = 5 (REC-SED), *n* = 6 (REC-EXR). **n** Soleus rate of force development across stimulation frequencies; independent one-tailed t-test, *n* = 5 (REC-SED), *n* = 6 (REC-EXR). **o** Soleus fatigue resistance during repeated contractions; independent one-tailed t-test, *n* = 5 (REC-SED), *n* = 6 (REC-EXR). **p** Partial least squares discriminant analysis (PLS-DA) of metabolite profiles from cecal content, serum, and muscle. **q** Mean decrease accuracy of top exercise-associated features. **r** Correlation of pipecolic acid-associated features. **s-t** Normalized abundance of pipecolic acid and succinate in cecal content, serum, and muscle; Tukey box plot (median, IQR, 1.5× IQR whiskers, outliers shown as individual points); independent two-tailed t-test, *n* = 9. Data are presented as mean ± SD for (**b**–l); mean ± SEM in **m**-**o**. All data presented are from biological replicates. Source data are provided in the Source Data file.

We performed exploratory cecal content transfers from sedentary and exercise-trained mice to exercise-naïve mice subjected to unilateral HLI in order to determine the effect of cecal transfers on skeletal muscle phenotype and, ultimately, identify exercise-associated microbial metabolites (Fig. 1a). First, we performed metagenomic sequencing to assess the impact of transfer on recipient microbial profiles. Total microbial abundance was significantly higher in REC-EXR versus REC-SED (*p* < 0.001) (Supplementary Fig. 1a). Alpha diversity was also higher in REC-EXR compared to REC-SED (*p* < 0.01) (Supplementary Fig. 1b). Principal component analysis with donor-recipient matching (three pooled donors per three recipients; see methods, Fig. 1a) revealed notable differences in beta diversity between REC-SED and REC-EXR groups, although recipients do not cluster tightly with their specific matched donors (Supplementary Fig. 1c). Bray-Curtis similarity was used to determine similarity within-match (i.e., recipient vs. matched donor), between-match (i.e., recipient vs. donors from same treatment group), and cross-group (i.e., recipient vs. donors from opposite treatment group) (Supplementary Fig. 1d). Bray-Curtis similarities within-match versus between-match were not significantly different (*p* = 0.483), indicating that recipients are not more similar to their specific matched donor than to other donors from the same treatment group. However, within-match versus cross-group similarity was significantly different (*p* < 0.01), showing that recipients are more similar to matched donors than to opposite-group donors. Between-match versus cross-group similarity was also highly significant (*p* < 0.001), confirming that differentiation between treatment groups is maintained. These analyses indicate that while matched donor-specific taxonomic recapitulation was not achieved, treatment-group-specific microbial characteristics were transferred such that recipient group microbial profiles diverge in a pattern indicative of donor influence.

The taxa abundance heat map further highlights these divergent microbial profiles between the two recipient groups (Supplementary Fig. 1E). Volcano plot analysis of differentially abundant species showed several significantly altered bacterial populations when comparing REC-EXR versus REC-SED (Supplementary Fig. 1f). Linear discriminant analysis (LDA) identified key predictive bacterial features distinguishing the recipient groups, with *Akkermansia muciniphila* showing the strongest association with the REC-EXR group (Supplementary Fig. 1g). Quantification confirmed significantly higher abundance of both *Akkermansia muciniphila* (*p* < 0.0001) (Supplementary Fig. 1h) and *Muribaculaceae bacterium DSM 103720* (*p* < 0.0001) (Supplementary Fig. 1i) in REC-EXR compared to REC-SED mice. For detailed metagenomic comparison between DON, SED, and EXR groups, see Supplementary Fig. 2–4.

Together, these findings suggest that the transfer of cecal content from sedentary and exercised hosts into exercise-naïve recipients successfully transfers treatment-group-specific microbial characteristics, resulting in distinct microbial communities between REC-SED and REC-EXR groups that mirror donor groups. Moreover, given the heightened differences in beta diversity between REC groups relative to differences between DON groups (Supplementary Fig. 1c), as well as the maintenance of alpha diversity and microbial abundance during transfer between EXR groups (Supplementary Fig. 1a, b, Supplementary Fig. 2b, c, 3b, c, 4b, c), these metagenomic data suggest that the differences in microbial profile between donor SED and EXR mice are accentuated following transfer.

### Cecal transfers from exercised donors ameliorate muscle loss

After seven cecal transfers over the course of five weeks, we induced muscle atrophy in exercise-naïve recipient mice through 10 days of unilateral HLI via casting using a 3-D printed cast adapted from Moore et al^10^. (Fig. 1a, Supplementary Fig. 5a). The 10-day time point was chosen due to its ability to induce consistent decreases in muscle mass and myofiber cross-sectional area (CSA) in the muscles of the posterior compartment of the lower leg (i.e., soleus, plantaris, and gastrocnemius muscles) (Supplementary Fig. 5b–f). The anterior compartment of the lower leg was not impacted by HLI, as there was no change in normalized tibialis anterior muscle mass or fiber CSA (Supplementary Fig. 5g–i), thus we focused our assessments on the muscles of the posterior compartment.

Prior to transfer, cecal contents were pooled from groups of three donors matched by total distance ran during the exercise protocol to eliminate exercise volume as a confounding factor (Fig. 1b). Soleus and plantaris (a slow- and fast-twitch muscle, respectively) mass were decreased in both REC-SED and REC-EXR groups following HLI (*p* < 0.0001) (Fig. 1c, d). The gross measurement of muscle mass may be confounded by various factors such as the precision of muscle excision, edema, and glycogen levels, which can obscure true changes in myofiber size. We therefore assessed muscle size via CSA, the gold standard for directly quantifying myofiber size. Mean soleus muscle fiber CSA was lower with HLI in both REC-SED (−42.7 ± 4.2%) and REC-EXR (−27.7 ± 13.9%) (*p* < 0.0001) (Fig. 1e), and average plantaris muscle fiber CSA was lower by −29.6 ± 14.7% (*p* < 0.01) and −20.3 ± 11.0% (*p* < 0.01), respectively (Fig. 1f). However, there was a significant interaction effect (*p* < 0.05) (Fig. 1e) between HLI and cecal transfer in the soleus such that skeletal muscle atrophy was 35.1% lower on average in REC-EXR compared to REC-SED (*p* < 0.01), an effect which is seen in the CSA of both Type I (42.6%; *p* < 0.05) and Type IIa (20.6%; *p* = 0.07) muscle fibers (Fig. 1g). There was a trend for a greater reduction in mean plantaris muscle fiber CSA (31.6%; *p* = 0.09) in REC-EXR, although this was not as apparent in the CSA of Type IIa (14.9%) or Type IIb/x muscle fibers (25.3%) (Fig. 1h). Other than a small decrease in the proportion of Type IIa muscle fibers in the REC-SED group following HLI, there were no changes in muscle fiber-type composition in either the soleus or plantaris muscles (Fig. 1i–l). These results demonstrate that cecal transfers from exercise-trained mice is able to blunt muscle atrophy in the slow-twitch soleus muscle, with more mild reductions in muscle atrophy of the fast-twitch plantaris muscle.

To assess whether the reduction in skeletal muscle atrophy affected muscle function, we performed ex vivo muscle function analyses of the soleus, the murine muscle most representative of human muscle in terms of fiber-type composition and transcriptome^11^, following HLI. The soleus was excised and placed in a myograph where it was stimulated at frequencies ranging from 10 to 120 Hz to assess strength, then stimulated at 60 Hz for 150 repeated contractions separated by 2 s to assess fatigue resistance. There were no significant differences in specific force generation at any stimulation frequency (Fig. 1m). However, the rate of force development (RFD) was higher across the stimulation frequencies in the REC-EXR (area under curve *p* < 0.05) (Fig. 1N), suggesting that the REC-EXR group had greater power. Moreover, REC-EXR maintained a higher force production relative to the first contraction during the fatigue protocol (area under curve *p* < 0.0001) (Fig. 1O), indicating that cecal transfers from the DON-EXR resulted in greater fatigue resistance in the soleus muscle. Together, these data demonstrate that the structural differences between REC-SED and REC-EXR were accompanied by improved muscle function in REC-EXR relative to REC-SED.

To identify candidate microbial-derived muscle-preserving metabolites conferred through cecal transfer, we performed untargeted metabolomics on cecal content, serum, and gastrocnemius muscle from REC-SED and REC-EXR. Partial Least-Squares Discriminant Analysis (PLS-DA) revealed distinct metabolite profiles between REC-SED and REC-EXR across all sample types (i.e., cecal, serum, muscle) (Fig. 1p), demonstrating that transfer of the exercise-trained gut microbiome alters peripheral tissues in recipient mice, even without exercise. Integrating metabolomic data from all sample types, we performed mean decrease in accuracy (MDA) analyses via random forest machine learning to determine predictive accuracy and metabolite importance. This analysis identified pipecolic acid, a lysine metabolite, as the top microbial-derived metabolite predictive of REC-EXR (Fig. 1q). Succinate, a TCA cycle intermediate, was among the top exercise-associated metabolites and correlated strongly with pipecolic acid (Fig. 1q, r), along with 2-oxindole-3-acetate. We previously reported succinate as a predicted post-biotic with positive effects on skeletal muscle bioenergetics, while acetate had minimal effects^7^.

To validate these findings, we performed targeted metabolomics in the cecal content and serum of mice treated with polyethylene glycol (PEG). By 24 hours post-PEG treatment, pipecolic acid abundance was reduced in both the cecal content (−69.4%, *p* < 0.01) and serum (−39.9%, *p* < 0.05) (Supplementary Fig. 6a, b). Initially, succinic acid decreased in the cecal content (−76.2%, *p* < 0.05 by independent t-test), but was elevated by 24 hours ( + 4,129.7%, *p* < 0.001) along with non-significant reductions in the serum (−50.9%, *p* = 0.13) (Supplementary Fig. 6c, d). Therefore, given the higher level of pipecolic acid and succinate in all sample types from REC-EXR (Fig. 1s, t) and the sensitivity of pipecolic acid and succinate to gut microbiome perturbations (Supplementary Fig. 6a–d), we sought to establish the efficacy of using microbial metabolites pipecolic acid and succinate to treat muscle atrophy.

### Microbial metabolites enhance muscle size and function during disuse

**Fig. 2:**
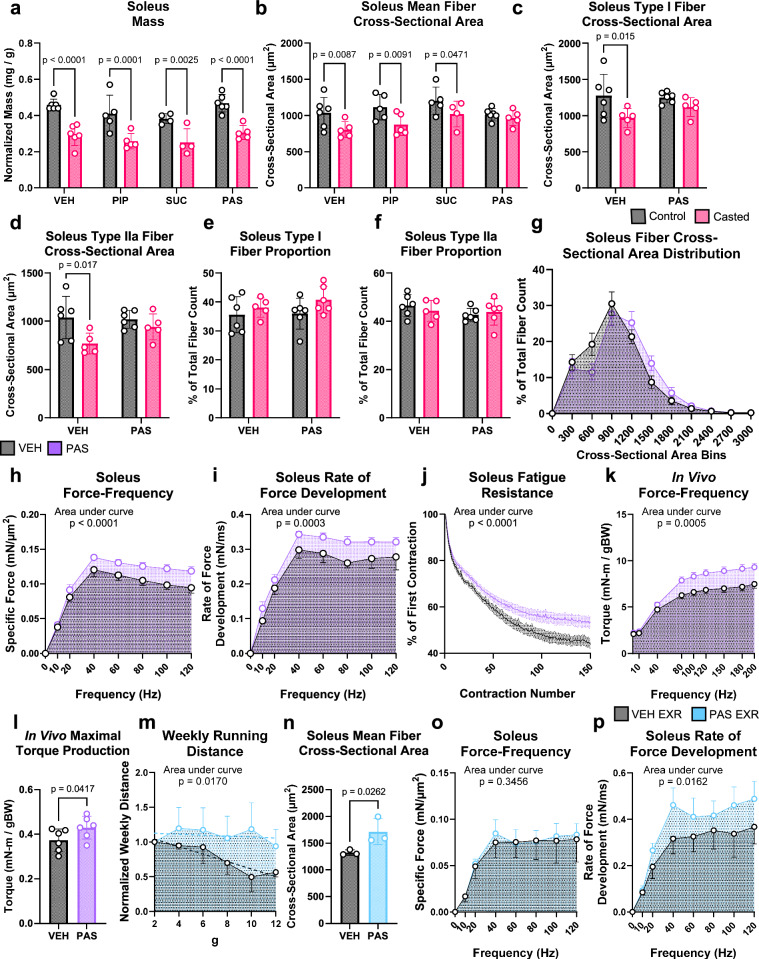
Microbial metabolites pipecolic acid and succinate potentiate the positive effects of cecal transfers on muscle size and function during disuse and promote exercise adaptation. **a** Soleus mass following 10-day hindlimb immobilization in vehicle (VEH), pipecolic acid (PIP), succinate (SUC), and pipecolic acid and succinate (PAS) groups; two-way ANOVA with Fisher’s post hoc. **b** Soleus mean muscle fiber cross-sectional area (CSA); two-way ANOVA with Fisher’s post hoc. **c**,**d** Soleus type I and IIa muscle fiber CSA; two-way ANOVA with Fisher’s post hoc. **e**,**f** Soleus type I and IIa muscle fiber proportions; two-way ANOVA with Fisher’s post hoc. **g** Soleus muscle fiber CSA distribution; *n* = 5 (VEH), *n* = 6 (PAS). **h** Ex vivo soleus force-frequency relationship; independent one-tailed t-test, *n* = 5 per group. **i** Soleus rate of force development across stimulation frequencies; independent one-tailed t-test, *n* = 5 per group. (**j**) Soleus fatigue resistance during repeated contractions; independent one-tailed t-test, *n* = 5 per group. **k** In vivo plantarflexor force-frequency relationship; independent one-tailed t-test, *n* = 6 per group. **l** In vivo plantarflexor maximal torque production; independent one-tailed t-test. (**m**) Weekly running distance during exercise with VEH or PAS relative to first week; independent one-tailed t-test, *n* = 3 per group. **n** Soleus mean muscle fiber CSA following exercise with VEH or PAS; independent one-tailed t-test. **o**,**p** Ex vivo soleus force-frequency relationship and rate of force development following exercise with VEH or PAS; independent one-tailed t-test, *n* = 3 per group. Data are presented as mean ± SD for **a**–**f**,**l**,**n**; mean ± SEM in **g**–**k**,**m**,**o**,**p**. All data presented are from biological replicates. Source data are provided in the Source Data file.

In order to determine the efficacy of purified microbial metabolites in conferring the benefits of exercise, we administered pipecolic acid and succinate via edible hydrogels, either separately (PIP and SUC, respectively) or in combination (PAS), for 10 days during HLI. Soleus muscle mass decreased in every group (*p* < 0.01 to 0.0001) (Fig. 2a); however, soleus mean muscle fiber CSA was maintained by PAS treatment (Fig. 2b) unlike vehicle (VEH) (*p* < 0.01), PIP (*p* < 0.01), and SUC groups (*p* < 0.05). We observed similar results in the plantaris muscle (Supplementary Fig. 7a–f). PAS treatment maintained both Type I and Type IIa CSA of the soleus muscle (Fig. 2c, d) with no changes in muscle fiber-type composition (Fig. 2e, f). Frequency histograms of soleus muscle fiber size revealed a shift towards larger fiber sizes in the PAS group (Fig. 2g).

Ex vivo muscle function analyses showed that PAS treatment resulted in significantly higher soleus strength (area under curve *p* < 0.0001), RFD (area under curve *p* < 0.0001), and fatigue resistance (area under curve *p* < 0.0001) relative to VEH (Fig. 2h–j). The effects of PAS on muscle function were more robust than those of EXR cecal transfers, demonstrating the direct administration of purified microbial metabolites potentiated the positive effects of EXR cecal transfers. Additionally, we performed in vivo muscle function analyses by stimulating the plantarflexors (i.e., soleus, plantaris, and gastrocnemius) at frequencies ranging from 10 to 200 Hz. PAS treatment preserved in vivo torque production at 80-200 Hz (*p* < 0.05; area under curve *p* < 0.0001) (Fig. 2k), as well as maximal torque production (*p* < 0.05) (Fig. 2l), indicating the preservation of the function of entire muscle groups (i.e., plantar flexors) consisting of slow- and fast-twitch muscles in their native environments. Collectively, these results demonstrate that microbial metabolites associated with an exercise-trained gut microbiome can prevent skeletal muscle atrophy and preserve muscle function in slow- and fast-twitch muscles when directly administered to mice undergoing HLI. Moreover, these data provide convincing evidence that the targeted administration of key microbiome-derived metabolites not only replicates the positive effects of cecal transfers from exercised donors, but also enhances the beneficial effects, demonstrating exciting therapeutic potential.

Given the remarkable ability of PAS administration to preserve muscle mass and function with disuse atrophy, we tested whether PAS treatment would improve exercise performance. Mice were administered either VEH or PAS or via drinking water during six weeks of exercise-training that was modified from our previously described progressive weighted wheel running (PoWeR) protocol^12^. Magnetic weights (2 g/week) were progressively added to the running wheel until the mice were running with an additional 12 g during the final week of the training period. Mice administered PAS maintained weekly running volume relative to the first week, while mice in the VEH group had progressively lower running volume (area under curve *p* < 0.05) (Fig. 2m). Mean soleus muscle fiber CSA was significantly larger in the PAS group (*p* < 0.05) (Fig. 2n), indicating a greater hypertrophic response to exercise training compared to VEH group. Thus, the enhanced hypertrophic growth of the PAS group likely reflected their higher training stimulus. We repeated the ex vivo muscle function analyses described above, finding that specific force was unchanged (area under curve *p* = 0.35) while RFD (area under curve *p* < 0.05) was higher in mice receiving PAS relative to those receiving VEH (Fig. 2o, p). Since the force data is normalized to CSA, it is not surprising that specific force did not change given the robust increase in CSA with PAS administration. Collectively, these results demonstrate that PAS administration not only prevented atrophy during disuse but promoted muscle hypertrophy by enhancing exercise performance.

### Pipecolic acid and succinate preserve ribosomal integrity

**Fig. 3:**
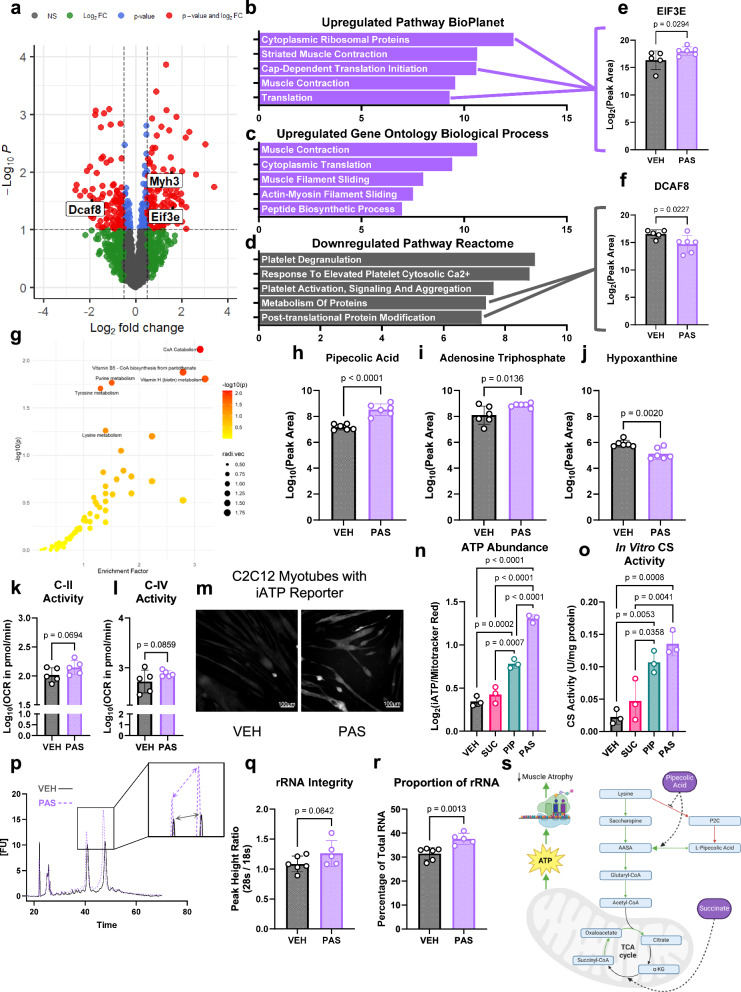
Microbial metabolites pipecolic acid and succinate stabilize cell energetics, reduce nucleotide degradation, and preserve ribosomal integrity. **a** Volcano plot of differentially expressed proteins in the gastrocnemius muscle between vehicle (VEH) and pipecolic acid and succinate (PAS); limma empirical Bayes moderated t-test, *n* = 5 (VEH), *n* = 6 (PAS). **b–d** Enrichment analysis of upregulated and downregulated pathways. **e**,**f** Protein abundance of eukaryotic translation initiation factor 3 subunit E (EIF3E) and DDB1 and CUL4 associated factor 8 (DCAF8); independent one-tailed t-test. **g** Pathway analysis of differentially expressed metabolites; mummichog permutation-based pathway enrichment, *n* = 6 per group. **h–j** Metabolite abundance of pipecolic acid, adenosine triphosphate (ATP), and hypoxanthine; independent one-tailed t-test. **k–l** Mitochondrial complex II/III and IV activity; independent one-tailed t-test. **m** C2C12 myotubes with ATP reporter (iATP). (**n**) ATP abundance in C2C12 myotubes treated with VEH, succinate (SUC), pipecolic acid (PIP), or PAS; one-way ANOVA with Tukey’s post hoc. **o** Citrate synthase (CS) activity in C2C12 myotubes; one-way ANOVA with Tukey’s post hoc. **p** Representative rRNA traces from VEH and PAS treated muscles. **q** rRNA integrity measured by 28S:18S ratio; independent one-tailed t-test. **r** Proportion of rRNA to total RNA; independent one-tailed t-test. **s** Proposed mechanism of action for PAS treatment in preventing muscle atrophy; created with BioRender.com. ASSA = L-2-aminoadipic acid 6-semialdehyde; P2C = Δ1-piperideine 2-carboxylate; α-KG = α-ketoglutarate. Data are presented as mean ± SD for **e**,**f**,**h**–**l**,**n**,**o**,**q**,**r**. All data presented are from biological replicates. Source data are provided in the Source Data file.

To elucidate the mechanism through which PAS treatment exerts its effects on skeletal muscle, we began by performing proteomics on the HLI gastrocnemius muscles from VEH and PAS treated groups. Proteomics analysis revealed 308 differentially expressed proteins (DEPs, *p* < 0.10): 169 upregulated and 139 downregulated (Fig. 3a). To interpret the biological and functional implications of these DEPs, we utilized Enrichr^13–15^ for gene ontology and pathways enrichment analysis (Fig. 3b–d). Upregulated DEPs showed significant enrichment in cytoplasmic ribosomal proteins, cap-dependent translation initiation, and translation (Fig. 3b). These included numerous structural ribosomal proteins (e.g., RPL3L, RPL4, RPL11, RPL17, RPL22, RPL23A, RPL29, RPL32, RPS16, RPS25), ribosome associated proteins (e.g., RPS6KA), and translation initiation factors (e.g., EIF3C, EIF3D, EIF3E). Notably, EIF3E, a subunit of eukaryotic initiation factor 3 (eIF3) crucial for maintaining sarcomeric structure in skeletal muscle^16^, was higher with PAS treatment (*p* < 0.05) (Fig. 3e). Moreover, upregulated DEPs were enriched for processes such as muscle/striated muscle contraction and muscle/actin-myosin filament sliding, including proteins such as MYH3, MYH4, MYH7, MYH8, TPM1, TPM2, TPM4, ACTA1, and ACTA2 (Fig. 3b, c). Downregulated DEPs were enriched for pathways involved in protein metabolism and post-translational protein modification (Fig. 3d). Notably, DCAF8, a known MuRF1 binding partner that promotes muscle atrophy through proteasomal degradation of sarcomeric proteins^17^, was downregulated (*p* < 0.05) (Fig. 3f). Taken together, these proteomic data suggest that PAS treatment mitigates atrophy by maintaining sarcomeric protein expression, potentially through enhanced protein synthesis and reduced protein degradation. Next, we assessed whether PAS influenced mTORC1 signaling as a means of preserving muscle size during atrophy. There were no differences in the total expression or phosphorylation level of AKT or p70S6K (Supplementary Fig. 8a–f; uncropped Western blot images provided in the source data file), indicating activation of mTORC1 signaling was not a component of PAS mechanism of action.

To further elucidate the effects of PAS treatment on skeletal muscle during atrophy, we performed untargeted metabolomics on the HLI gastrocnemius muscles from VEH and PAS treated groups. There were clear distinctions between VEH and PAS treated groups (Supplementary Fig. 9a). Pipecolic acid was the top elevated metabolite in the PAS group (Supplementary Fig. 9b), confirming successful delivery to skeletal muscle. Pathway analysis of differentially expressed metabolites indicated enrichment of the lysine breakdown pathway, of which pipecolic acid is involved, as well as CoA and purine metabolism pathways in the PAS group (Fig. 3g). Along with higher pipecolic acid levels (*p* < 0.0001) (Fig. 3h), we observed significantly higher levels of ATP with PAS treatment (*p* < 0.05) (Fig. 3i), suggesting increased energy availability. Moreover, hypoxanthine, an adenine nucleotide degradation product, was lower in PAS-treated muscle (*p* < 0.01) (Fig. 3j). This is noteworthy as increased adenine nucleotide degradation is associated with skeletal muscle atrophy^18^. We assessed mitochondrial respiration, finding that the activity of mitochondrial complexes II (*p* = 0.07) and IV (*p* = 0.09) (Fig. 3k–l) were trending higher in the PAS group, despite no change in the expression of any of the 5 OXPHOS complexes (Supplementary Fig. 10a–e; uncropped Western blot images provided in the source data file).

To determine if PAS treatment was acting directly on muscle cells to elevate ATP levels, we treated C2C12 myotubes with PAS and delivered a fluorescent ATP reporter^19^ (iATP) via a muscle specific adeno-associated virus (MyoAAV)^20,21^. We validated the reporter in vitro by treating myotubes with 2-deoxy-glucose (Supplementary Fig. 11a). Next, we treated iATP+ myotubes with VEH, PIP, SUC, or PAS for three days (Supplementary Fig. 11b) and quantified fluorescence to determine ATP levels. PAS treatment increased ATP levels more than VEH, SUC or PIP (*p* < 0.0001) (Fig. 3m, n). The elevation in ATP level was accompanied by higher citrate synthase activity in PIP (*p* < 0.01) and PAS (*p* < 0.001) relative to VEH (Fig. 3o).

Given that up to 90% of cellular RNA is rRNA^22^, lower hypoxanthine levels with PAS treatment suggests that PAS might be reducing rRNA degradation. To assess RNA integrity, we measured the ratio of 28S to 18S rRNA^23^ of gastrocnemius muscle from VEH and PAS treated mice (Fig. 3p). The 28S to 18S rRNA ratio was trending (*p* = 0.06) higher in PAS-treated muscle (Fig. 3q), suggesting lower levels of rRNA degradation. Additionally, the proportion of rRNA relative to total RNA was significantly higher in PAS relative to VEH (*p* < 0.01) (Fig. 3r) indicating higher translational capacity. Ribosome biogenesis is an energy-intensive process, and rRNA transcription is tightly coupled to cellular energy status^24,25^. The higher levels of ribosomal proteins combined with the metabolomic and rRNA data suggest that PAS treatment may preserve skeletal muscle size and function through the enhancement of cellular energy status and translational capacity.

## Discussion

This study identified novel microbial metabolites with therapeutic potential for preventing skeletal muscle atrophy. Using exploratory cecal content transfers from exercise-trained donors combined with integrative multi-omics analysis, we identified pipecolic acid and succinate as key exercise-associated microbial metabolites. Direct administration of these metabolites prevented disuse-induced atrophy and preserved muscle function, demonstrating their therapeutic activity independent of gut microbial transfer. These findings establish pipecolic acid and succinate as promising therapeutic candidates and provide proof-of-concept that exercise-associated microbial metabolites can serve as exercise mimetics for treating exercise-responsive conditions.

Previous studies have demonstrated the ability of an exercise-trained microbiome to confer the benefits of exercise to exercise-naïve hosts^26,27^. Our findings support these data and demonstrate that an exercise-trained gut microbiome can elicit phenotypic effects on adult skeletal muscle consistent with exercise preconditioning^28^. Likewise, microbial products have been shown to elicit therapeutically relevant effects on host tissues, including skeletal muscle^9,29–32^. Our work specifically identifies microbial products from an exercise-trained microbiome which confer the benefits of exercise phenotypically (i.e., prevent atrophy during disuse) and functionally (i.e., preserve muscle strength, power, and fatigue resistance), thus indicating widespread therapeutic potential and setting the stage for novel drug discovery.

An emerging concept supported by the findings of this study is that the benefits of regular exercise are mediated by exercise-induced changes to the gut microbiome. Studies have assessed the effectiveness of microbial transplants from exercise donors in altering glucose metabolism^26,33,34^, obesity^26,33,34^, colitis^27^, and exercise capacity^35,36^. By transplanting microbiota from exercise-responsive and non-responsive humans into mice, Liu et al^26^. demonstrated that the metabolic signature of the donors was replicated in the recipient mice. This study showed the potential of conferring the metabolic benefits of exercise through the gut microbiome. We establish that cecal transfers from exercise-trained donors affect recipient skeletal muscle in a manner consistent with exercise adaptation, resulting in clinically relevant improvements in muscle function. Our work further demonstrates the exciting potential of leveraging an exercise-trained gut microbiome to confer the benefits of exercise, a therapeutic avenue which could significantly impact populations with exercise limitations.

While transplanting microbiomes from exercised donors has therapeutic potential in itself, the primary goal of this study was to leverage cecal transfers as a discovery platform for identifying candidate metabolites with therapeutic potential. The fact that purified metabolites administered orally recapitulated and, in some measures, exceeded the protective effects observed with cecal transfers demonstrates that these specific compounds, rather than complex microbial community structure, mediate the therapeutic benefits. This finding has important translational implications, as chemically defined compounds can be standardized, dosed precisely, and administered without the variability inherent in live microbial preparations. Recently, there have been several such microbial-derived metabolites identified with positive effects on skeletal muscle. Urolithin A, a compound derived from the microbial metabolism of ellagitannins, has been shown to improve strength and exercise performance through the regulation of mitophagy in skeletal muscle^29^. Oral supplementation of urolithin A showed promise in improving strength and performance in humans through the improvement of mitochondrial function^9^, although the results vary^37^. More recently, it was reported that nicotinic acid derived from *Bifidobacterium adolescentis* improved mitochondrial function in skeletal muscle, leading to positive effects on sarcopenia^5^. By leveraging exercise to modify the gut microbiome, then identifying related metabolites, we demonstrate the ability to confer the benefits of exercise through the direct administration of microbial-derived, exercise-responsive metabolites.

In the present work, pipecolic acid and succinate were identified as key exercise-responsive microbial metabolites. When administered together, these metabolites prevented the loss of skeletal muscle size and function during atrophic conditions. Both pipecolic acid^38^ and succinate^39^ have been suggested to impact mTORC1 signaling in vitro, although we showed that administration of PAS had no effect on the activation of key proteins in the mTORC1 signaling pathway (i.e., AKT, p70S6K) in skeletal muscle in vivo. Interestingly, other groups have suggested that pipecolic acid rescues the harmful effects of an inflammatory challenge through the inhibition of mTORC1 signaling in bone marrow-derived macrophages^40^. Instead, our work demonstrates promising effects of PAS treatment on ATP levels and mitochondrial complex activity. Succinate has been shown to rescue mitochondrial dysfunction in various tissues^41–43^, including skeletal muscle^44^. While the effect of pipecolic acid on mitochondrial function has not been elucidated, pipecolic acid, as a product of lysine catabolism, can be metabolized into acetyl-CoA and enter the TCA cycle^45^. It may be that the microbial-driven increase in pipecolic acid and succinate in skeletal muscle in response to exercise (or direct administration of these metabolites) increases substrate flux through the TCA cycle, thereby promoting ATP generation. Ribosome biogenesis is an energy-intensive process^24,25^, so this maintenance of energy status may be linked to our findings that PAS preserves rRNA content and the expression of ribosomal proteins. Given the negative influence of atrophic conditions on ATP production and ribosomal/translational capacity^46–48^, this preservation of ribosomal integrity through the promotion of ATP production may explain how PAS is able to prevent skeletal muscle atrophy and preserve muscle function. The ribosome has been postulated as a small-molecule sensor in microorganisms^49^ and plants^50^, so it may be that PAS affects the ribosome through direct interaction. Therefore, the present work provides several promising avenues for establishing the precise mechanism of PAS treatment in preventing disuse-induced atrophy in skeletal muscle (Fig. 3s). If indeed PAS impacts ribosomes directly, to the authors knowledge, this would be the first study demonstrating a microbial-derived metabolite to preserve mammalian host ribosomal abundance.

Several limitations of this study should be noted. Our work utilized cecal transfers to explore potential exercise-responsive microbial metabolites which were subsequently identified and administered directly. Cecal transfers did not result in matched donor-specific taxonomic recapitulation, and therefore our metagenomic conclusions remain limited. Moreover, our experiments were conducted in mice, and translating these findings to humans will require further investigation. However, pipecolic acid has been shown to increase with exercise in both mice and humans^40^, suggesting that the increase in microbial production of this metabolite is conserved in humans. Additionally, while we identified two key exercise-responsive microbial metabolites, the exercise-trained microbiome likely produces a complex mixture of beneficial metabolites that may act synergistically. Future studies should explore the broader metabolite profile and potential interactions, as well as focus on establishing the effects of exercise-responsive microbial metabolites on other tissues affected by exercise (e.g., heart, liver, brain). Nevertheless, the implications of these findings are far-reaching. Given the widespread benefits of exercise for conditions such as cardiovascular disease, Alzheimer’s disease, aging, and diabetes, our results provide a new avenue for the development of therapeutic interventions to treat exercise-responsive conditions. By leveraging an exercise-trained microbiome to identify key metabolites, we may be able to confer some exercise benefits to individuals unable to perform physical activity, such as bed-ridden patients or those with mobility limitations. Additionally, direct metabolite administration allows us to circumvent the gut microbiome entirely, which may be particularly relevant in patients receiving broad-spectrum antibiotics. Future research should focus on elucidating the precise mechanisms by which PAS treatment ameliorates disuse atrophy, exploring potential synergies between different exercise-responsive microbial metabolites, and establishing the efficacy of an exercise-trained gut microbiome in treating various disease models. Moreover, translational studies will be crucial to determine the applicability of these findings in human populations. In summary, this study opens new possibilities for microbiome-based therapies inspired by exercise, potentially revolutionizing our approach to treating muscle wasting and other exercise-responsive conditions.

## Methods

### Mice

Female C57BL/6 J mice (strain #000664; 4- to 12-month-old) were used for all experiments. Female mice were selected for this study due to their superior wheel running performance and predictable running behavior compared to their male counterparts^6,51,52^. Mice were allowed to acclimate to the university vivarium for >4 weeks to allow the gut microbiome to stabilize^53^. For experiments involving the gut microbiome transfers, mice were randomly assigned to one of the following groups: sedentary donor (DON-SED), exercised donor (DON-EXR), recipient from sedentary donor (REC-SED), or recipient from exercised donor (RED-EXR) (*n* = 9). During metabolite administration experiments, mice were randomly assigned to receive vehicle (VEH), succinate (SUC), pipecolic acid (PIP), or a combination of both succinate and pipecolic acid (PAS) (*n* = 3-6). Sample sizes align with previous work interrogating the gut-muscle axis in mice^2,5–8^. All groups had *ad libitum* access to feed (2918, 18% protein diet; Inotiv, West Lafyette, IN) and water during the duration of the study unless otherwise noted and were monitored daily to ensure animal welfare. All experiments were conducted using the same groups of mice with the exception of ex vivo muscle function and in vivo muscle function experiments which required additional cohorts. All animal procedures were performed according to national and local guidelines and approved by the Institutional Animal Care and Use Committee at the University of Kentucky.

### Exercise training

Donor mice were subjected to 8 weeks of progressive weighted wheel running (PoWeR) as described previously^12^. Briefly, mice were singly housed in running wheel cages with free access to the running wheel for one week of acclimation. After acclimation, weights consisting of 2 g in week 1, 3 g in week 2, 4 g in week 3, 5 g in weeks 4 and 5, and 6 g in weeks 6-8 were added to one side of the wheel. Sedentary mice were singly housed under the same conditions with a locked running wheel.

For experiments involving VEH and PAS treatment during exercise training (*n* = 3; 12-month-old female mice), mice were singly housed in running wheel cages as described above. After acclimation, weights consisting of 2 g in week 1, 4 g in week 2, 6 g in week 3, 8 g in week 4, 10 g in week 5, and 12 g in week 6 were added to one side of the wheel. All running data (distance in km) were acquired via ClockLab software (Actimetrics, Wilmette, IL).

### Cecal transfers

The transfer of cecal contents to a new host has recently emerged as a successful method of transplanting the gut microbiome in murine models^54,55^. Cecal contents were collected from exercised and sedentary donor mice, resuspended into sterile PBS at a concentration of 150 mg/ml, and stored at −80 °C. Prior to the first transfer, recipient mice were treated with polyethylene glycol (PEG) (SLBZ3934; Sigma-Aldrich, St. Louis, MO) to clear the existing microbiome^56^. Mice received four boluses of 200 µL of 425 g/L PEG via oral gavage over the course of one hour. Cecal contents collected from nine exercise-trained or sedentary mice were pooled in groups of three (randomly assigned for sedentary donors, matched by running volume for exercised donors; Fig. 1B) and transferred via oral gavage (200 µL per transfer) to anesthetized recipient mice six hours after the final PEG treatment. Recipient mice received transfers on three consecutive days, followed by a single transfer per week for the next four weeks, resulting in seven total transfers over the course of five weeks (Fig. 1A). Following the final transfer, mice were unilaterally cast for 10 days to induce skeletal muscle atrophy (Supplementary Fig. 5, see below).

### Microbial metabolite administration

For experiments involving the direct administration of microbial metabolites, mice received either VEH (gel only; *n* = 6), SUC (S7501; Sigma-Aldrich; *n* = 5), PIP (P2519; Sigma-Aldrich; *n* = 5), or PAS via MediGel Sucralose hydrogels (74-02-5022; ClearH_2_O, Westbrook, ME) (*n* = 6). Mice received free access to the hydrogels for 10 days during hindlimb immobilization. During this period, mice consumed an average of 112.3 ± 26.3 mg/kg/d and 222.8 ± 51.4 mg/kg/d of succinate and pipecolic acid, respectively.

For experiments involving the administration of VEH and PAS during six weeks of exercise training, mice received free access to VEH (2% sucrose) or PAS (146.0 ± 10.9 mg/kg/d succinate, 292.0 ± 21.7 mg/kg/d pipecolic acid [50-194-7204; Fisher Scientific, Waltham, MA], 2% sucrose) through their drinking water.

### Unilateral hindlimb immobilization

A model of hindlimb immobilization utilizing casting was developed^10,57^ and subsequently modified by our lab using 3-D printing technology. This model allows the contralateral leg to remain mobile and serve as a control, thus limiting inter-animal variation in muscle size. Briefly, the leg is encapsulated in a plastic, 3-D printed external cast which maintains the leg in gentle flexion at the knee and dorsiflexion at the ankle. The casts were secured via twisty ties to prevent escape. Mice were casted for 10 days in all immobilization experiments.

### Ex vivo muscle function

All protocols for ex vivo muscle function were adapted from Minchew et al^58^. Mice were anesthetized via isoflurane and euthanized via cervical dislocation. The soleus of the casted leg was excised and mounted to a force transducer in an organ bath (820MO; Danish Myo Technology, Essen, Germany) in Krebs Ringer buffer (in mM: 119 NaCl, 5.0 KCl, 5.0 NaHCO_3_, 1.25 CaCl_2_, 1.0 KH_2_PO_4_, 10 HEPES, 1.0 MgSO_4_; pH 7.2) via tendon clamps and maintained at room temperature. The proximal tendon was secured to the force transducer, while the distal tendon was secured to a length dial (0.1 mm increments). Following a 10-minute equilibration period, optimal length (L_O_) was determined by adjusting the muscle length by increments of 0.1–0.5 mm to determine maximal force output in response to 20 V, 20 ms pulse width stimulations delivered using parallel platinum electrodes and separated by 30 seconds of rest (CS4+ Stimulator; Danish Myo Technology). The muscle was then stimulated every 3 min at L_O_ at 10, 20, 40, 60, 80, 100, and 120 Hz (biphasic, 300 ms trains, 0.2 ms pulse width, 20 V) to generate force-frequency curves. Following another 3-minute rest period, a fatigue resistance protocol was performed where the muscle was subjected to 150 repeated 60 Hz stimulations separated by 2 seconds (same parameters above). Afterwards, L_O_ was recorded, the muscle was blot dried, weighed, covered in Tissue-Tek O.C.T. Compound (4583; Sakura Finetek, Torrance, CA), and frozen in liquid nitrogen-cooled isopentane in preparation for immunohistochemistry. Force output was recorded using LabChart software (v.8.1.19) and analyzed using MATLAB (ver. R2024b Update 5). Absolute force output (mN) was normalized to cross-sectional area (CSA) determined via immunohistochemistry (see below) and reported as specific force (mN/µm^2^). Rate of force development (RFD) was calculated as follows: RFD = Δ Force/Δ Time from the beginning of the contraction to peak force. Samples from which reliable force curves were unable to be produced were excluded.

### In vivo muscle function

Plantarflexion in vivo isometric peak torque was assessed as we previously described^59,60^. Briefly, anesthetized mice were placed in the supine position on a 37 °C temperature regulated platform (809c in situ mouse apparatus, Aurora Scientific, Aurora, ON, Canada) and the hindlimb was secured at the knee with the foot taped to a dual-mode lever and motor (300D-300C-LRFP, Aurora Scientific) so that the tibia was parallel to the platform and the knee was held at a 90° angle. Percutaneous needle electrodes placed lateral to the knee were used to stimulate the tibial nerve with an electrical stimulator (High Power Bi-Phase Stimulator, Aurora Scientific). To maximize torque production and eliminate activation of dorsiflexors, needles were adjusted in response to repeated twitches to identify the optimal placement. Peak tetanic torque was recorded at 10, 40, 80, 120, 150, 180, and 200 Hz stimulations (0.25 s duration, 50 mA) using DMC v6.000 and analyzed with MATLAB. Data are reported as normalized to body mass (mN-m/gram of BW).

### Tissue collection

Following immobilization, hindlimb musculature (soleus, plantaris, and gastrocnemius) from both lower hindlimbs was carefully excised, weighed, and either covered in Tissue-Tek O.C.T. Compound and snap-frozen in liquid nitrogen-cooled isopentane in preparation for immunohistochemistry (soleus, plantaris), or directly snap-frozen in liquid nitrogen for downstream molecular analyses (gastrocnemius). For SED and EXR experiments specifically, cecal contents and serum were also collected at sacrifice. Cecal contents were immediately snap frozen in liquid nitrogen. To obtain serum, blood was collected, allowed to clot, and centrifuged at 150 x *g* for 15 min. The supernatant was removed and centrifuged again at 300 x *g* for 15 min. Quadriceps muscles were also collected from the ex vivo muscle function cohorts of VEH and PAS mice for complex activity analyses.

### Immunohistochemistry

Muscle fiber-type staining procedures were carried out as previously described^61^. Briefly, unfixed soleus sections were incubated overnight with antibodies against myosin heavy chain types I (BA.D5), IIA (SC.71), and IIB (BF.F3) (1:100; Developmental Studies Hybridoma Bank, Iowa City, IA), as well as rabbit anti-laminin IgG (1:100; L9393; Sigma-Aldrich). The sections were then incubated with fluorescence-conjugated secondary antibodies against the various mouse immunoglobulin subtypes. Muscle sections were imaged using either a Zeiss upright fluorescent microscope (Zeiss AxioImager M1 Oberkochen, Germany) or an Olympus BX61VS Upright Fluorescent Microscope (Evident Scientific, Bethlehem, PA) at 20x magnification. MyoVision 2.0, an unbiased automated image analysis program developed by our lab, was used to determine myofiber CSA in a blinded manner^62,63^. Samples from which reliable CSA and/or fiber-type were unable to be determined (i.e., high variability in technical replicates, section damage) were excluded.

### Western blots

Gastrocnemius samples were homogenized using a THb Handheld Tissue Homogenizer and Hard Tissue Omni Tip Plastic Homogenizer Probes (Omni International, Kennesaw, GA) in RIPA Lysis and Extraction Buffer (786-490; G-Biosciences, St. Louis, MO) with Halt Protease & Phosphatase Inhibitor Cocktail (1861281; ThermoFisher Scientific, Waltham, MA). For quantification of protein abundance, samples were resolved on a 4–12% Bis-Tris Plus Protein Gels (NW04120BOX; ThermoFisher Scientific) and transferred to a PVDF membrane (LC2002; ThermoFisher Scientific). The membrane was blocked with 5% BSA in 0.1% Tween Tris-buffered saline (TBS-T) and incubated at room temperature with primary antibodies against AKT (9272S; Cell Signaling), p-AKT (4051S, Ser473; Cell Signaling), p70S6K (9202S; Cell Signaling), p-p70S6K (9206S, Thr389; Cell Signaling), and mitochondrial complex proteins (45-8099; ThermoFisher Scientific). Blots were developed using enhanced chemiluminescence (Clarity Western ECL Substrate; Bio-Rad, Hercules, CA) and imaged on a Chemidoc imaging system (Bio-Rad).

### Metagenomics

Microbial DNA was extracted from the cecal contents as previously described^6^. Briefly, upon euthanasia, the cecum was excised from DON-SED, DON-EXR, REC-SED, and REC-EXR mice and the contents were carefully removed and immediately frozen in liquid nitrogen. DNA was isolated from cecal content using the PureLink Microbiome DNA purification kit (A29790; ThermoFisher Scientific) according to the manufacturer’s instructions. Samples were sent to Alkek Center for Metagenomic and Microbiome Research (Baylor College of Medicine, Houston, TX) for metagenomic sequencing. Samples were analyzed on a HiSeq X Ten Sequencing System (Illumina, Sandiego, CA). Taxonomic classification was performed with MetaPhlan 3.0^64^. One REC-SED sample was excluded due to low read depth. Microbial diversity, profiling, and differential statistics analyses were performed and visualized using the online software MicrobiomeAnalyst 2.0^65^ and R (version 4.3.2)^66^ package Vegan^67^: Chao1 indices were used to determine alpha diversity; principal component analyses (PCoA) based on Bray-Curtis distance were used to determine beta diversity; linear discriminant analyses (LED) were used to identify predictive features; DESeq2 package was used to identify differential microbes. One sample was removed from the SED-EXR group due to low read counts.

### Untargeted metabolomics

Cecal content, serum, and gastrocnemius samples from SED and EXR mice were sent to Metabolon, Inc. (Morrisville, NC) for untargeted metabolite profiling. Samples were analyzed on a ThermoFisher Scientific Q-Exactive spectrometer interfaced with a heated electrospray ionization source and Orbitrap mass analyzer. Metabolites were separated on an ACQUITY UPLC Peptide BEH C18 column (100 mm×2.1 mm, 1.7 µm particle size; 186003555; Waters, Milford, MA). Compounds were identified using Metabolon’s internal library of authenticated standards. Partial least squares discriminant analysis (PLS-DA) and random forest classification were performed using the online software MetaboAnalyst 6.0^68^.

For VEH and PAS experiments, gastrocnemius samples were pulverized (CryoPREP CP02 Cryogenic Dry Pulverization; Covaris, Woburn, MA), suspended in 1 mL of extraction solution (50% HPLC-grade methanol and 1% formic acid), and homogenized using zirconium silicate beads (ZSB10; Next Advance, Inc., Troy, NY) in a Bullet Blender Gold (Next Advance, Inc.). Supernatant was filtered using Captiva EMR-Lipid cartridges (5190-1002; Agilent Technologies, Santa Clara, CA) placed on top of autosampler vials (5190-2280; Agilent Technologies). Samples were then dried and concentrated using a Savant SpeedVac Integrated Vacuum Concentrator System and Kit (SPD2030; ThermoFisher Scientific) at 25 °C until a complete pellet was formed ( ~ 48 h). The pellet was resuspended in 5% acetonitrile and 0.1% formic acid, filtered (0.22 µm; 8160; Costar, Glendale, AZ), and stored in polymer feet autosampler inserts (5183-2088; Agilent Technologies) in autosampler vials. Metabolites were separated using a SeQuant ZIC-pHILIC column (150 mm×2.1 mm, 5 µm particle size; 150454; Sigma-Aldrich). Mobile phase A consisted of 10 mM ammonium acetate in water, pH 9.8 and mobile phase B consisted of 100% methanol. All chemicals were LC-MS grade. The column flow rate was set to 0.15 mL/min, column temperature set at 25 °C. Metabolites were separated over a 19-minute gradient from 90% B to 30% B. The column was washed for 5 minutes at 30% B, and then re-equilibrated at 90% B for 8 minutes. Samples were analyzed on a ThermoFisher Scientific Orbitrap Exploris 240 mass spectrometer coupled to a Vanquish Neo UHPLC system (ThermoFisher Scientific). Polarity-switching MS1 only acquisition was acquired for each polarity set at 120,000 FWHM resolution and a scan range was set to 80-1200 Daltons; the automatic gain control (AGC) target was set to ‘Custom’ at 1e6 absolute AGC value and a maximum inject time of 50 ms. Spectra were analyzed using Compound Discoverer Software 3.3SP (ThermoFisher Scientific) and searched against ChemSpider databases (BioCyc, Human Metabolome, and KEGG) to identify compound names. PLS-DA, differential statistics, and pathways enrichment analyses were performed and visualized using MetaboAnalystR R package^69^.

### Targeted metabolomics

Cecal content and serum were collected from mice treated with PEG according to the methods described above at 0 or 24 hours post treatment (*n* = 2). Samples were cyropulverized (SPEX SamplePrep, Metuchen, NJ), suspended in 1:10 of 100% methanol (Supelco, Bellefonte, PA), filtered and dried as described above, and reconstituted in 80/20 (v/v) acetonitrile/water. Metabolites were separated using a Poroshell 120 HILIC-Z column (150 mm×2.1 mm, 2.7 µm particle size; Agilent Technologies). Mobile phase A consisted of 10 mM ammonium acetate in water with 2.5 μM Agilent InfinityLab deactivator additive, pH 9.0 and mobile phase B consisted of 10 mM ammonium acetate in water/acetonitrile 15:85 (v:v) with 2.5 μM InfinityLab deactivator additive, pH 9.0. All chemicals were LC-MS grade. The column flow rate was set at 0.4 mL/min, column temperature at 25 °C, and injection volume at 10 µL. Samples were analyzed on an Agilent 6495c iFunnel QQQ mass spectrometer coupled to an Agilent 1290 Infinity II HPLC system (Agilent Technologies). Spectra were analyzed using Agilent MassHunter Quantitative Analysis, ver. 12 (Agilent Technologies). Reference standards (P45850, PHR1418; Sigma-Aldrich) were used for metabolite quantification.

### Proteomics

Gastrocnemius muscles were suspended in RIPA buffer (786-490; G-Biosciences, St. Louis, MO) and homogenized using an Omni Tissue Homogenizer (Omni International, Kennesaw, GA). After a 10-minute incubation on ice, samples were centrifuged at 10,000 x *g* for 10 minutes twice. Pierce BCA Protein Assay kits (23225; ThermoFisher Scientific) were used to quantify protein levels. Equivalent protein amounts (20 µg) were enzymatically digested using the EasyPep MS Sample Prep Kits (A40006; ThermoFisher Scientific) according to the manufacturer’s protocol. Peptides were resuspended in 5% acetonitrile and 0.1% formic acid and analyzed on a ThermoFisher Scientific Orbitrap Exploris 240 mass spectrometer with an EASY-Spray source housing and a Vanquish Neo UHPLC system. Peptides were separated on an EASY-Spray HPLC column (150 mm×75 µm, 2 µm particle size; ES904; ThermoFisher Scientific) paired with a PepMap Neo 5μm C18 300μm x 5 mm Trap Cartridge (174500; ThermoFisher Scientific). Mobile phase A consisted of 0.1% formic acid in water and mobile phase B consisted of 0.1% formic acid in acetonitrile. The flow rate was set to 0.3 µL/min. Peptides were separated over a 90-minute linear gradient from 2-55% mobile phase B. The column was equilibrated for 1.5 min at 2% mobile phase B. Mobile phase B was increased to 10% over 10 minutes and increased again to 25% over the next 35 minutes. Mobile phase B was increased to 35% over 25 mins followed by 55% mobile phase B for 15 minutes. Following peptide separation, the column was washed at 0.75 µL/min at 98% mobile phase B for 8 minutes, followed by column re-equilibration at 2% mobile phase B for 5 minutes. Data was collected using a data-dependent acquisition (DDA) strategy. The instrument was set to 60,000 resolution, with a top N precursor ions in a 3 second cycle time. Data was collected using positive ionization including charge states 2-4. Full scan (MS1) settings were set as follows: (a) scan range: 375-1200 m/z, (b) RF lens (%): 45, (c) AGC target: custom, (d) normalized AGC target (%) 250, (e) maximum injection time mode: custom, and (f) maximum injection time (ms): 20. Peptide fragmentation spectra (MS2) were collected using a normalized HCD collision energy set to 26%, 2 m/z isolation window, 15,000 resolution, normalized AGC target set to 50%, and automatic maximum injection time. Data were analyzed against the Mus musculus UniProt database (downloaded March 2024) using the ThermoFisher Scientific Proteome Discoverer (version 3.1) software and the SEQUEST search algorithm. Proteomic output was analyzed using the R (version 4.3.2)^66^ package DEP^70^. One outlier sample was removed from the VEH group. Data were visualized using the EnhancedVolcano package^71^.

### Electron transport chain complex activity

The activity of mitochondrial electron transport chain Complexes II and IV was assessed as described previously^72^ using frozen quadriceps samples. Samples were homogenized in Mitochondrial Isolation Buffer (MIB) (in mM: 70 sucrose, 220 mannitol, 5 KH_2_PO_4_, 5 MgCl_2_, 1 EGTA, 2 HEPES, 3 mM BSA; pH 7.4) using a THb Handheld Tissue Homogenizer and Hard Tissue Omni Tip Plastic Homogenizer Probes (Omni International, Kennesaw, GA). Mitochondria were isolated by differential centrifugation, and protein concentration was determined using a BCA Protein Assay Kit (71285-3; Sigma-Aldrich). 20 µg of protein was loaded into Seahorse XF24 Cell Culture Microplate (100777-004; Agilent Technologies) with 500 µL Seahorse Assay Buffer (103575-100, Agilent Technologies) with 2 mg/100 mL cytochrome C and spun at 2000 g for 20 minutes with no break at 4 °C to adhere mitochondria to the plate. Oxygen consumption rate (OCR) was measured in a Seahorse XF24 Analyzer (Agilent) at baseline and after each of the following injections: 1 mM succinate and 2 μM rotenone; 4 μM antimycin A; 0.5 mM N,N,N’,N’-tetramethyl-p-phenylenediamine (TMPD) and 1 mM ascorbic acid (freshly prepared), pH 7.4; 50 mM sodium azide. Complex II (C-II) activity was reported as the OCR recording after succinate and rotenone addition (prior to antimycin A injection). Complex IV (C-IV) activity was determined by subtracting the OCR after sodium azide addition from that after ascorbic acid + TMPD.

### rRNA integrity

RNA was isolated from gastrocnemius samples from VEH and PAS groups. Samples were homogenized in TRI Reagent (T9424; Sigma-Aldrich) with zirconium silicate beads (ZSB10; Next Advance, Inc.) using a Bullet Blender Gold (Next Advance, Inc.). After a 10-minute incubation on ice, samples were centrifuged at 10,000 x *g* for 10 min. RNA was isolated using Direct-zol RNA Miniprep Plus Kits (R2070; Zymo Research, Irvine, CA) according to manufacturer’s instructions. Samples were diluted 1:200 and run on an Agilent 2100 Bioanalyzer (Agilent Technologies) using RNA Pico Chips (5067; Agilent Technologies) to assess rRNA quality.

### In vitro iATP reporter

An ATP-GFP reporter (iATP) (Plasmid #209651; Addgene) was co-transfected with the capsid (MyoAAV 2 A) and helper (pAdDeltaF6, Plasmid #112867, Addgene) plasmids into HEK293T-17 cells using PEI Prime™ (AQ100, Serochem, Bloominton, MN). Viral particles were purified using iodixanol gradient ultracentrifugation and titered by ddPCR using primer/probe set targeting the ITR (TaqMan custom assay, ThermoFisher Scientific). Mouse C2C12 myoblasts (American Type Culture Collection, ATCC, Manassas, VA) were cultured in high glucose containing Dulbecco’s Modified Eagle growth medium (DMEM; 10-013-CM; Corning, Corning, NY) supplemented with 10% (*v*/*v*) fetal bovine serum (FB-73; Alkali Scientific) and 100 U/mL penicillin and 100 µg/mL streptomycin (15-140-148, ThermoFisher Scientific). Cells were infected with iATP-MyoAAV at an estimated 10^3^, 10^4^, and 10^5^ multiplicity of infection (MOI) in differentiation medium containing high glucose DMEM and 2% (*v*/*v*) horse serum (HS, H1138, Sigma-Aldrich) for 7 days. Cells were imaged using Olympus APEXVIEW APX100 (Evident Scientific) at 20x magnification. To confirm iATP reporters dynamically report ATP levels in cells, we subjected cells to filter sterilized Tyrode’s solution (pH 7.4) containing 1 mM MgCl_2_, 1.8 mM CaCl_2_, and 5.5 mM 2-deoxy-d-glucose, which essentially removes glucose from becoming a source of energy inside the cells thereby depleting cellular ATP abundance (Supplementary Fig. 11a). Reintroduction of differentiation media containing glucose increased cellular ATP levels overnight but does not return cells to pre-deoxy-glucose levels (Supplementary Fig. 11a).

For metabolite treatment experiments, mouse C2C12 myoblasts were differentiated and infected with iATP-MyoAAV at 10^3^ MOI (Supplementary Fig. 11b). After 7 days of infection, cells were treated with VEH, PIP (10 mM), SUC (2 mM), or PAS (10 mM and 2 mM, respectively) in differentiation media for 72h and imaged as described above. Cells were treated with 25 nM of MitoTracker Red (M7512; ThermoFisher Scientific) for 30 min and washed to control for mitochondrial abundance. Data represents iATP fluorescence intensity normalized to MitoTracker Red fluorescence intensity.

### Citrate synthase activity

Citrate synthase activity in cell homogenates was determined using an enzymatic assay as previously described^7,73^. Briefly, samples were mixed in a buffer containing Tris (100 mM, pH 8.0), 5,5’-Dithiobis-(2-nitrobenzoic acid) (DTNB) (10 mM) (D8130; Sigma Aldrich), acetyl CoA (30 mM) (A2056; Sigma Aldrich), and oxaloacetic acid (OAA) (10 mM) (O4126; Sigma Aldrich). Equal amounts of protein (8 µg) were loaded, and enzymatic activity was assessed by measuring the change in absorbance at 412 nM over 10 minutes using a Cytation 5 Multimode reader (Biotek, Winooski, VT). The following calculation was used to determine enzymatic activity: U/mg protein = (Δ Absorbance/Δ Time in minutes) / (13.6 × 0.625 × mg protein).

### Statistics

All statistical analyses were performed using GraphPad Prism version 10.2.3 for Windows (GraphPad Software, La Jolla, CA). Independent, one-tailed t-tests were used to test for differences in total microbial abundance, individual microbe abundance, alpha diversity, muscle mass, muscle CSA, torque production, protein and metabolite expression, mitochondrial complex activity, in vivo citrate synthase activity, and rRNA integrity and proportion between respective groups. One-sample t-tests were used to test differences between control and casted muscle CSA in 5, 7, and 10-day immobilized mice. Two-way ANOVAs or mixed-effect analyses (exercise, transfer; treatment, casting) with Fisher’s post hoc analyses were used to test for differences in alpha diversity, muscle mass, muscle CSA, muscle fiber proportion, and pipecolic acid and succinate levels in cecal content and serum within and between respective groups. Area under the curve was calculated for all force/torque-frequency, RFD, and fatigue curves and statistically significant differences were tested using independent one-tailed t-tests. Multiple, independent two-tailed t-tests were used to test differences in pipecolic acid and succinate abundances between SED and EXR groups in cecal content, serum, and muscle. One-way ANOVAs with Tukey’s post hoc analyses maximizing power were used to test for differences in donor running volume, ATP abundance, and citrate synthase activity in vitro between metabolite treated groups.

### Reporting summary

Further information on research design is available in the Nature Portfolio Reporting Summary linked to this article.

## Supplementary information

Supplementary Information

Reporting Summary

Transparent Peer Review file

## Source data

Source Data

## Data Availability

Raw metagenomics data generated in this study have been deposited in the NCBI BioProject database under the accession code PRJNA1457569. Raw proteomics data generated in this study have been deposited to the ProteomeXchange Consortium via the Proteomics Identifications Database under the accession code PXD077599. Raw metabolomics data generated in this study have been deposited in the MetaboLights database under the accession code MTBLS14386. Source data for all other experiments are provided with this paper. Source data are provided with this paper.
