# Supplementary material for: Exercise-associated microbial metabolites prevent skeletal muscle atrophy in adult female mice
Source: Nat Commun. 2026 Jul 10;17:8512. doi: 10.1038/s41467-026-74852-w (PMC13482395; doi:10.1038/s41467-026-74852-w)
Supplement: Supplementary file 1 [file 41467_2026_74852_MOESM1_ESM.pdf]

SUPPLEMENTARY INFORMATION

TITLE: Exercise-Associated Microbial Metabolites Prevent Skeletal Muscle Atrophy in Adult Mice

AUTHORS: Benjamin I. Burke[1,2†], Taylor R. Valentino[3†], Ahmed Ismaeel[4], Salim S. El-Amouri[1,2], Jensen Goh[1,2], Logan N. Scott[1,2], Bonnie J. Walton[1,2,5], Jai K. Joshi[1,2], Cecily R. Wood[1], Abigail Burrows-Franco[1,6], John B. May[7], Lance A. Johnson[1,8], Michael D. Flythe[9,10], Yuan Wen[1,2,5]✉, John J. McCarthy[1,2]✉

AFFILIATIONS:

[1]Department of Physiology, College of Medicine, University of Kentucky, Lexington, KY
[2]Center for Muscle Biology, College of Health Sciences, University of Kentucky, Lexington, KY
[3]Buck Institute for Research on Aging, Novato, CA
[4]Department of Anatomy, Physiology and Pharmacology, College of Veterinary Medicine, Auburn University, Auburn, AL[5]Division of Biomedical Informatics, Department of Internal Medicine, College of Medicine, University of Kentucky, Lexington, KY
[6]Mass Spectrometry and Proteomics Core, University of Kentucky, Lexington, KY
[7]Department of Plant and Soil Sciences, College of Agriculture, Food and Environment, University of Kentucky, Lexington, KY
[8]Sanders Brown Center on Aging, University of Kentucky, Lexington, KY
[9]USDA Agriculture Research Service, Forage-Animal Production Research Unit, University of Kentucky, Lexington, KY
[10]Department of Animal and Food Sciences, College of Agriculture, Food and Environment, University of Kentucky, Lexington, KY

†These authors contributed equally; ✉email: ywen2@uky.edu, jjmcca2@uky.edu

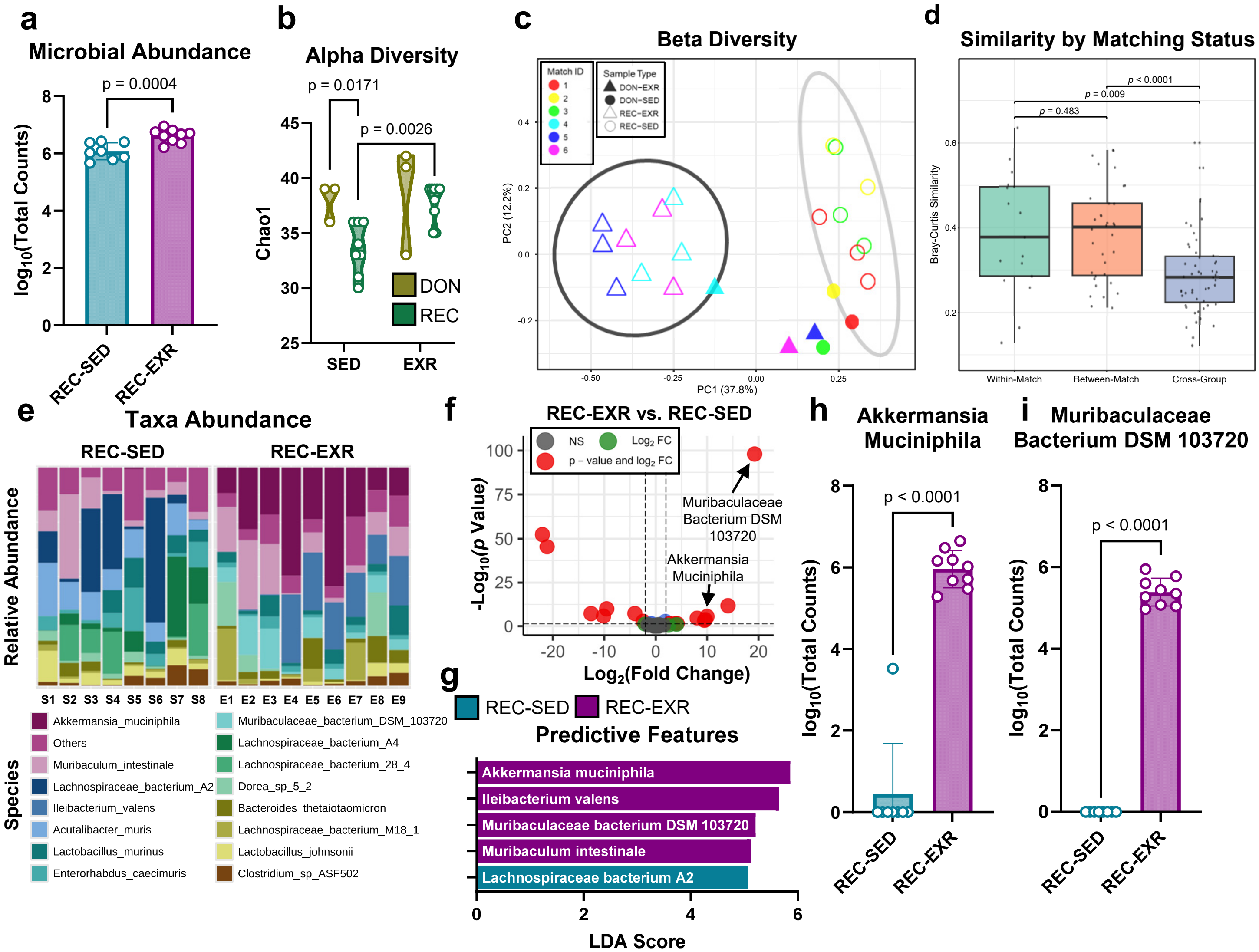

Supplementary Figure 1. Cecal Transfers from Exercise-Trained Donors Increase Microbial Abundance and Diversity in Exercise-Naïve Recipient Mice. (**a**) Microbial abundance in recipients of sedentary donor (DON) cecal transfers (REC-SED) and recipients of exercised donor cecal transfers (REC-EXR); independent one-tailed t-test. (**b**) Alpha diversity (Chao1) in DON and REC groups; two-way ANOVA with Fisher's post hoc. (**c**) Beta diversity shown by principal coordinates analysis (PCoA). (**d**) Bray-Curtis similarity within-match (i.e., recipient vs. matched donor), between-match (i.e., recipient vs. donors from same treatment group), and cross-group (i.e., recipient vs. donors from opposite treatment group); Tukey box plot (median, IQR, 1.5× IQR whiskers, outliers shown as individual points); one-way ANOVA with Tukey's post hoc, n = 3 (DON-SED, DON-EXR), 8 (REC-SED), 9 (REC-EXR). (**e**) Heat map of relative bacterial abundance in REC-SED and REC-EXR mice. (**f**) Volcano plot showing differentially abundant species between REC-EXR vs. REC-SED; DESeq2 Wald test, n = 8 (REC-SED), 9 (REC-EXR). (**g**) Linear discriminant analysis (LDA) score of predictive bacterial features. (**h-i**) Abundance of *Akkermansia muciniphila* and *Muribaculaceae bacterium DSM 103720* in recipient groups; independent one-tailed t-test. Data are presented as mean ± SD for **a**-**b**, **h**-**i**. All data presented are from biological replicates. Source data are provided in the Source Data file.

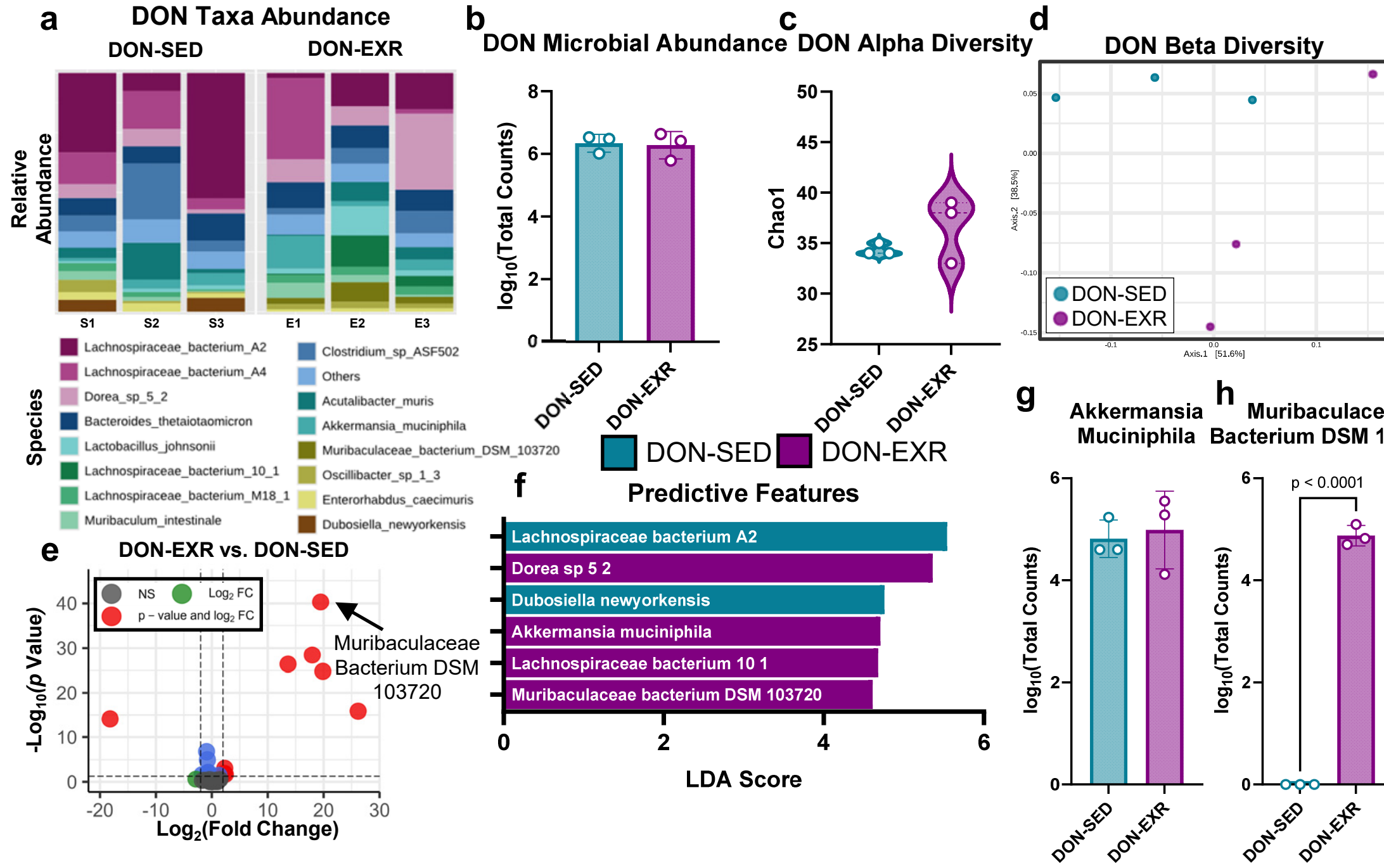

Supplementary Figure 2. Comparison of DON-SED and DON-EXR Microbial Profiles. (**a**) Heat map of relative bacterial abundance of sedentary donors (DON-SED) and exercised donors (DON-EXR). (**b**) Microbial abundance of DON groups; independent one-tailed t-test. (**c**) Alpha diversity of DON groups; independent one-tailed t-test. (**d**) Beta diversity of DON groups. (**e**) Volcano plot showing differentially abundant species between DON-EXR vs. DON-SED; DESeq2 Wald test, n = 3. (**f**) Linear discriminant analysis (LDA) score of predictive bacterial features. (**g-h**) Abundance of *Akkermansia muciniphila* and *Muribaculaceae bacterium DSM 103720* in DON groups; independent one-tailed t-test. Data are presented as mean ± SD for **b-c**, **g-h**. All data presented are from biological replicates. Source data are provided in the Source Data file.

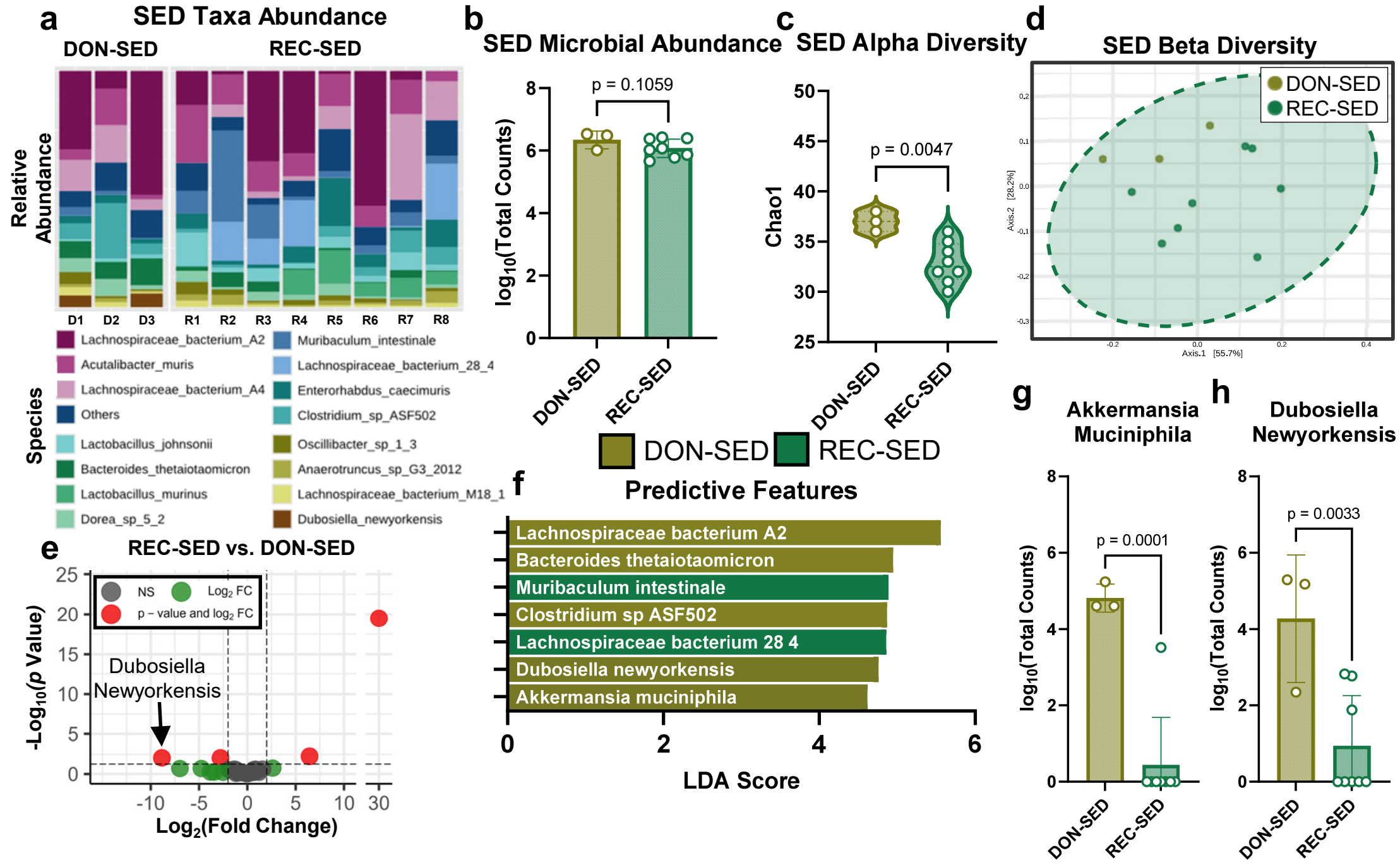

Supplementary Figure 3. Comparison of DON-SED and REC-SED Microbial Profiles. (**a**) Heat map of relative bacterial abundance of sedentary donors (DON-SED) and recipients of sedentary donor cecal transfers (REC-SED). (**b**) Microbial abundance of SED groups; independent one-tailed t-test. (**c**) Alpha diversity of SED groups; independent one-tailed t-test. (**d**) Beta diversity of SED groups. (**e**) Volcano plot showing differentially abundant species between REC-SED vs. DON-SED; DESeq2 Wald test, n = 3 (DON-SED), 8 (REC-SED). (**f**) Linear discriminant analysis (LDA) score of predictive bacterial features. (**g-h**) Abundance of *Akkermansia muciniphila* and *Dubosiella newyorkensis* in SED groups; independent one-tailed t-test. Data are presented as mean ± SD for **b-c**, **g-h**. All data presented are from biological replicates. Source data are provided in the Source Data file.

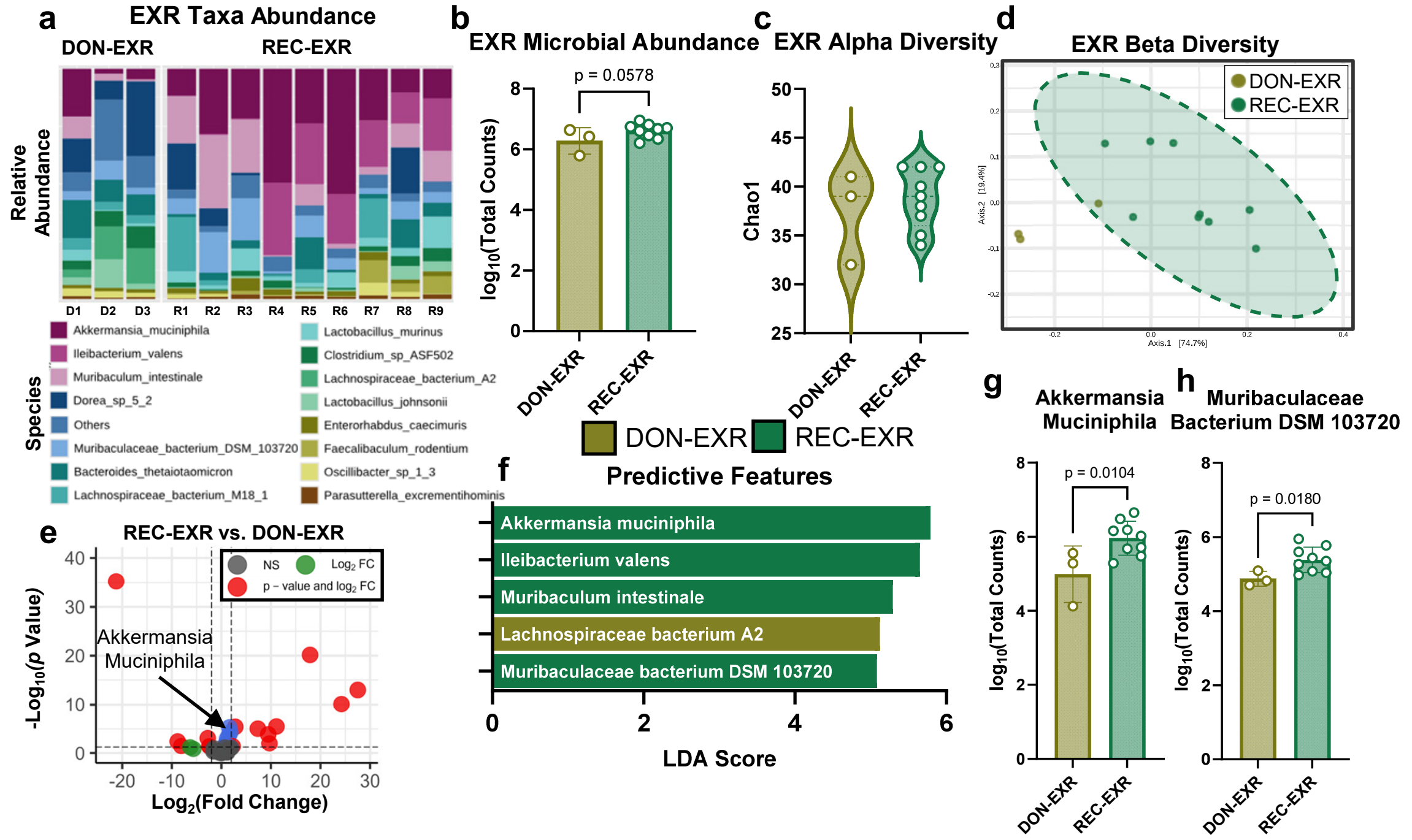

Supplementary Figure 4. Comparison of DON-EXR and REC-EXR Microbial Profiles. (**a**) Heat map of relative bacterial abundance exercised donors (DON-EXR) and recipients of exercised donor cecal transfers (REC-EXR). (**b**) Microbial abundance of EXR groups; independent one-tailed t-test. (**c**) Alpha diversity of EXR groups; independent one-tailed t-test. (**d**) Beta diversity of EXR groups. (**e**) Volcano plot showing differentially abundant species between REC-EXR vs. DON-EXR; DESeq2 Wald test, n = 3 (DON-EXR), 9 (REC-EXR). (**f**) Linear discriminant analysis (LDA) score of predictive bacterial features. (**g-h**) Abundance of *Akkermansia muciniphila* and *Muribaculaceae bacterium DSM 103720* in EXR groups; independent one-tailed t-test. Data are presented as mean ± SD for **b**-**c**, **g**-**h**. All data presented are from biological replicates. Source data are provided in the Source Data file.

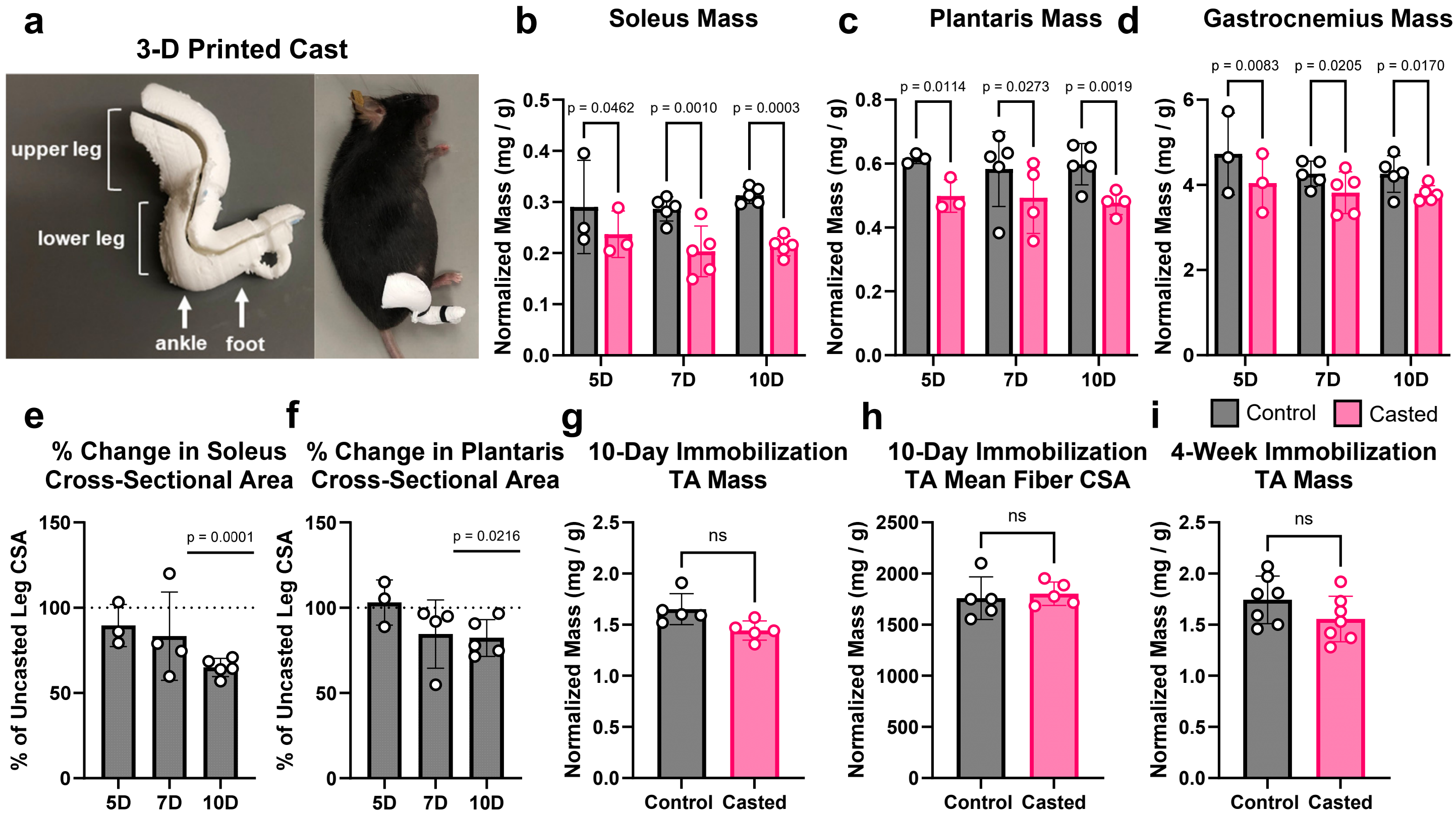

Supplementary Figure 5. Validation of the Hindlimb Immobilization Model. (**a**) 3D-printed cast design and application. (**b-d**) Muscle mass of soleus, plantaris, and gastrocnemius at 5, 7, and 10 days of immobilization normalized to body weight; two-way ANOVA with Fisher's post hoc. (**e-f**) Percent change in soleus and plantaris mean muscle fiber cross-sectional area (CSA) at 5, 7, and 10 days of immobilization; one-sample t-test. (**g-i**) Tibialis anterior (TA) muscle mass and CSA following 10 days and 4 weeks of immobilization; independent two-tailed t-test. Data are presented as mean ± SD. All data presented are from biological replicates. Source data are provided in the Source Data file.

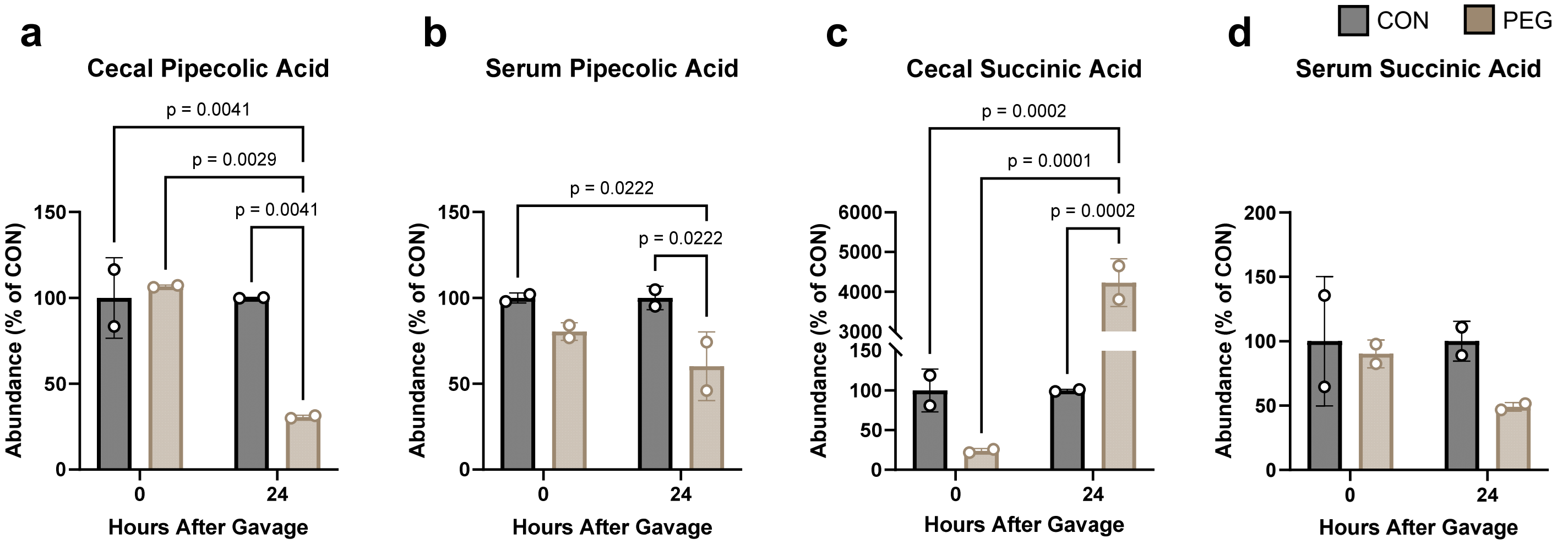

Supplementary Figure 6. Pipecolic Acid and Succinate Respond to Gut Microbiome Perturbations. Levels of (**a-b**) pipecolic acid and (**c-d**) succinic acid in the cecal content and serum of control (CON) and polyethylene glycol (PEG) treated mice 0 and 24 hours after treatment; two-way ANOVA with Fisher's post hoc. Data are presented as mean ± SD. All data presented are from biological replicates. Source data are provided in the Source Data file.

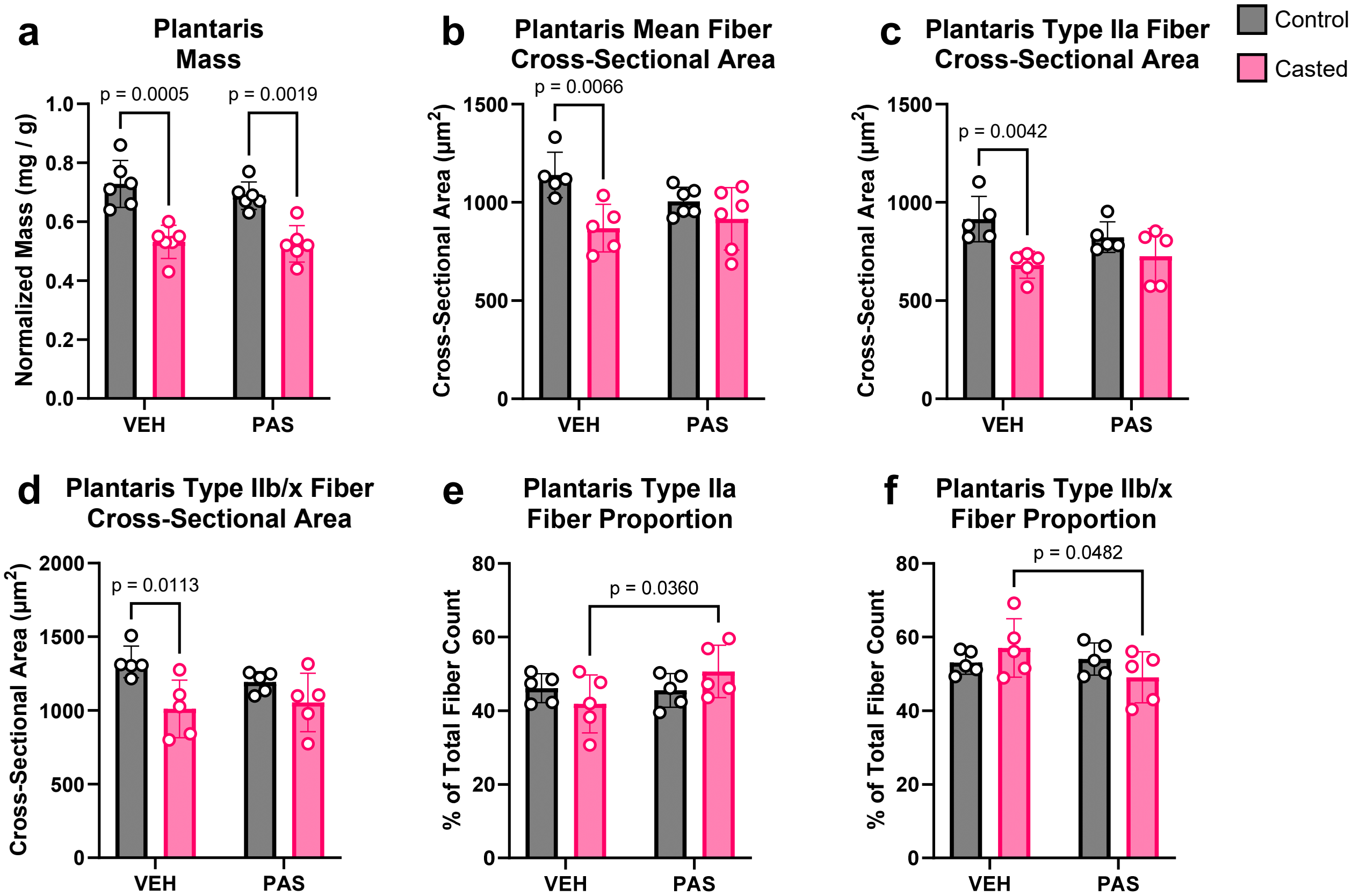

Supplementary Figure 7. Plantaris Muscle Mass, Fiber Size, and Fiber Type with Pipecolic Acid and Succinate Treatment. (**a**) Plantaris mass following 10-day hindlimb immobilization in vehicle (VEH) and pipecolic acid and succinate (PAS) groups; two-way ANOVA with Fisher's post hoc. (**b**) Plantaris mean muscle fiber cross-sectional area (CSA); two-way ANOVA with Fisher's post hoc. (**c-d**) Plantaris type IIa and IIb/x muscle fiber CSA; two-way ANOVA with Fisher's post hoc. (**e-f**) Plantaris type IIa and IIb/x muscle fiber proportions; two-way ANOVA with Fisher's post hoc. Data are presented as mean ± SD. All data presented are from biological replicates. Source data are provided in the Source Data file.

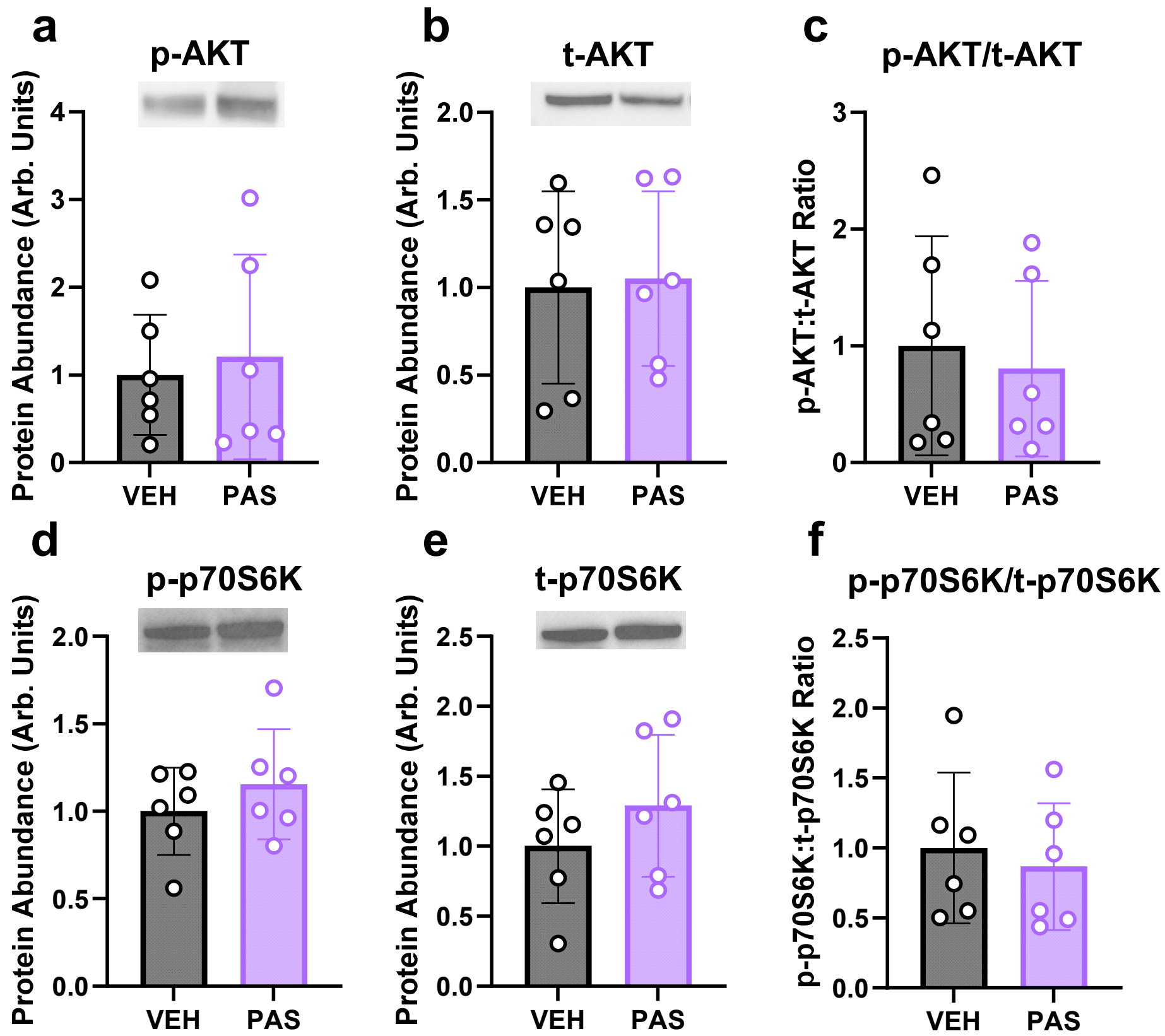

Supplementary Figure 8. Effects of Pipecolic Acid and Succinate Treatment on mTORC1 Signaling. Protein abundance and phosphorylation of (**a-c**) AKT and (**d-f**) p70S6K in the gastrocnemius of mice treated with vehicle (VEH) or pipecolic acid and succinate (PAS); independent one-tailed t-test. Data are presented as mean ± SD. All data presented are from biological replicates. Source data are provided in the Source Data file.

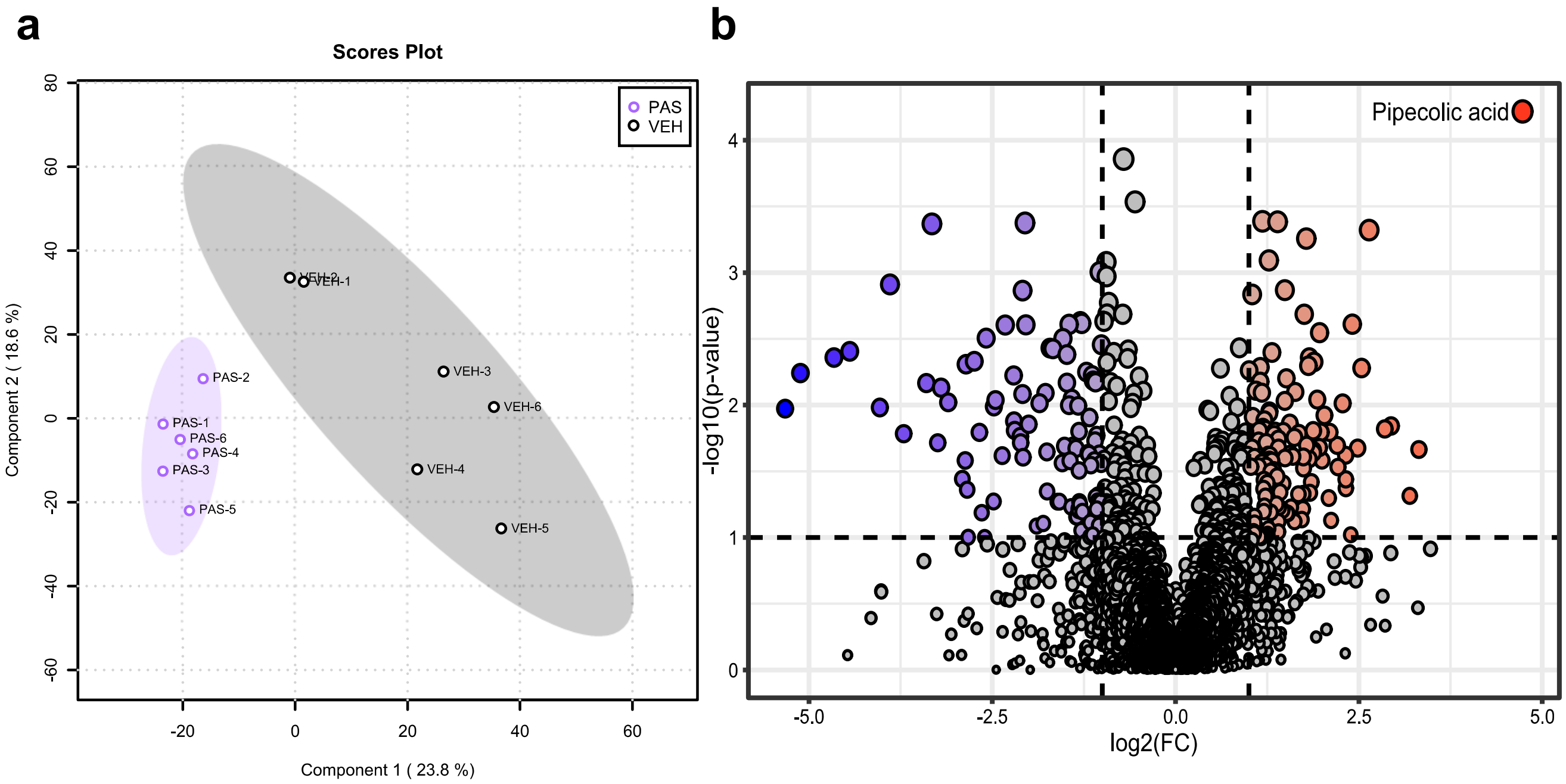

Supplementary Figure 9. Metabolomic Profiling of Skeletal Muscle from Pipecolic Acid and Succinate-Treated Mice. (**a**) Partial least squares discriminant analysis (PLS-DA) plot and (**b**) volcano plot of differentially expressed metabolites in the gastrocnemius of mice treated with vehicle (VEH) or pipecolic acid and succinate (PAS); independent two-tailed t-test; n = 6. All data presented are from biological replicates. Source data are provided in the Source Data file.

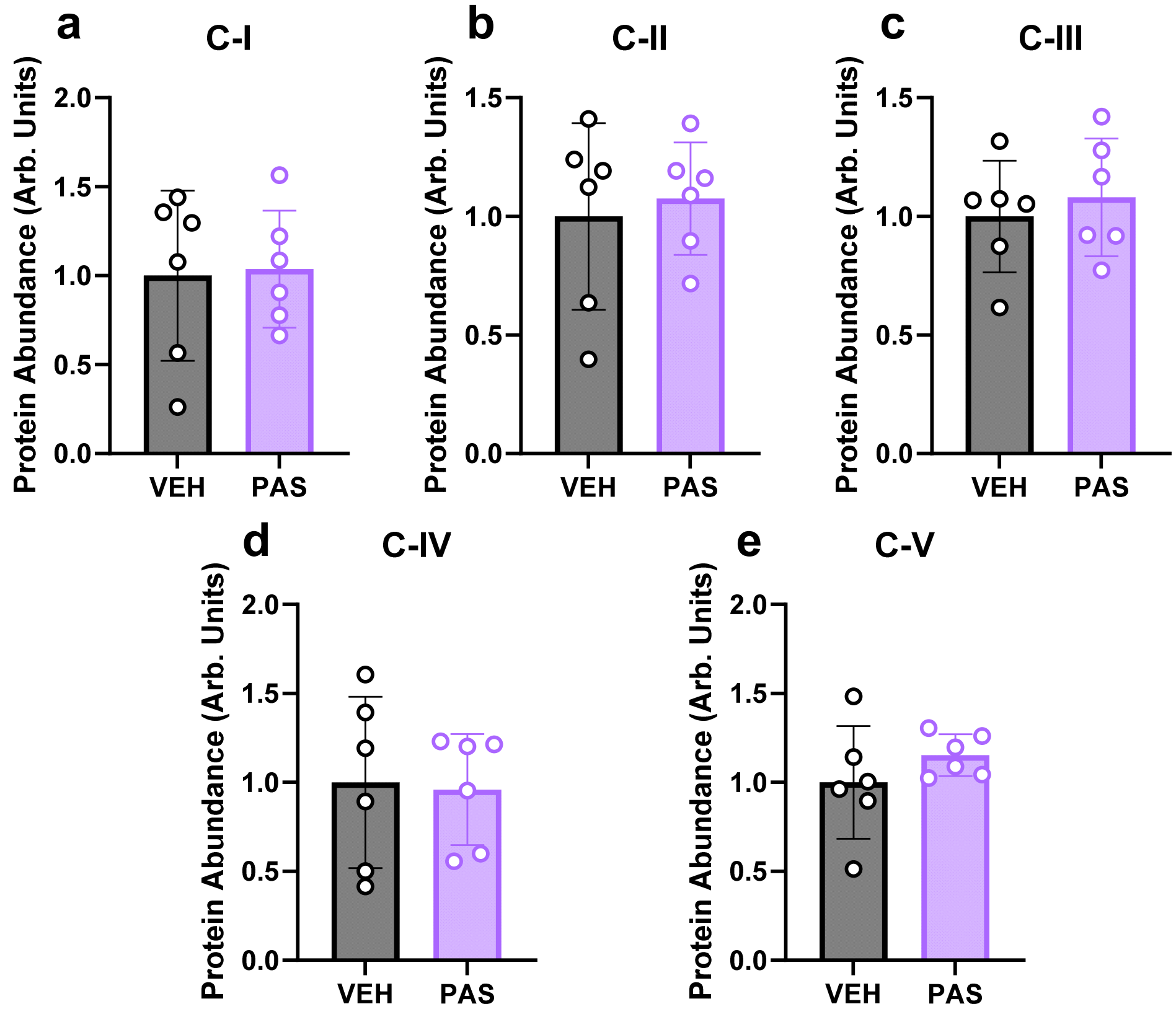

Supplementary Figure 10. Mitochondrial Complex Protein Expression Following Pipecolic Acid and Succinate Treatment. (**a-e**) Protein abundance of mitochondrial complexes I-V in the gastrocnemius of mice treated with vehicle (VEH) or pipecolic acid and succinate (PAS); independent one-tailed t-test. Data are presented as mean ± SD. All data presented are from biological replicates. Source data are provided in the Source Data file.

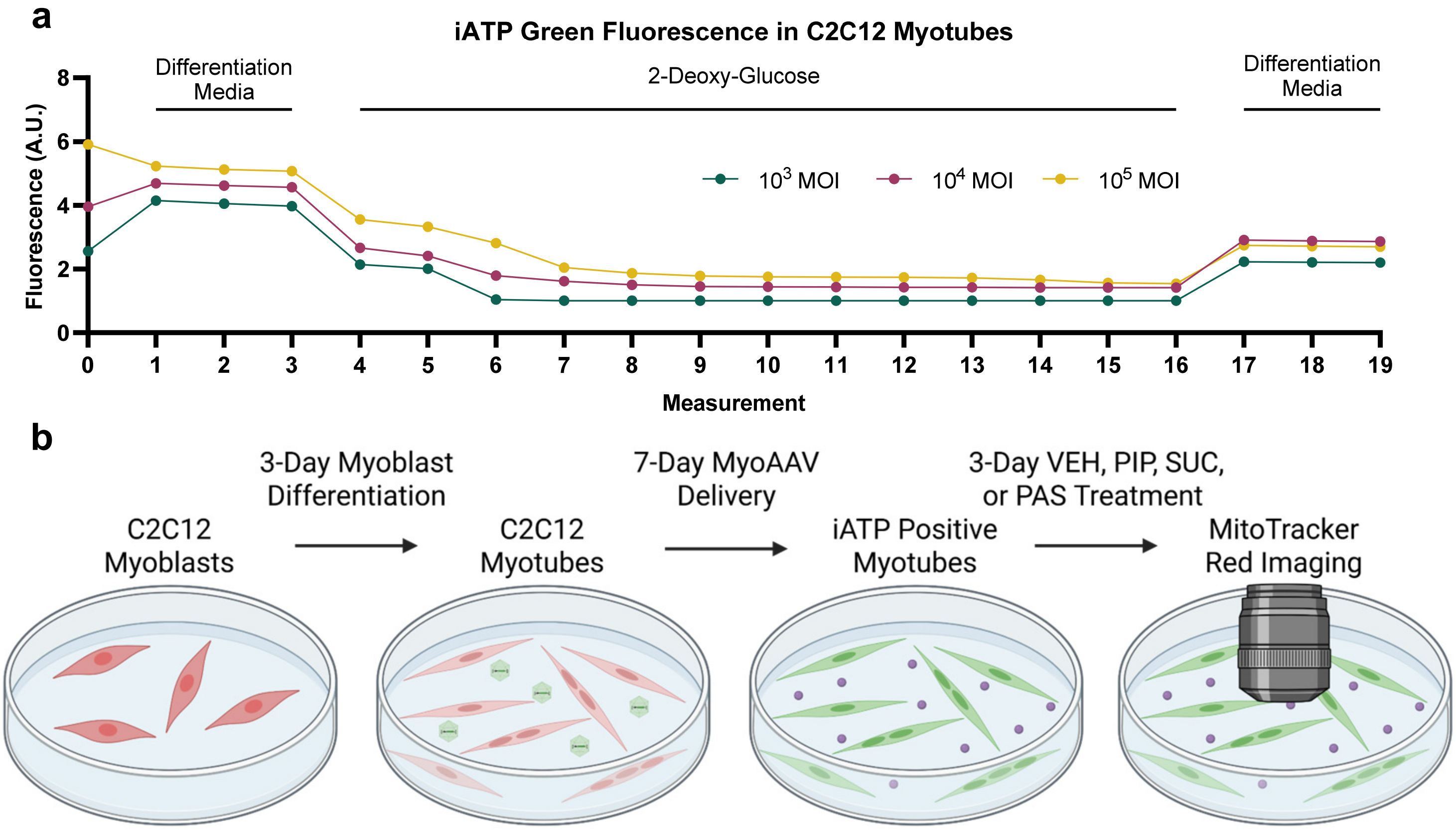

Supplementary Figure 11. ATP Reporter Validation. (**a**) iATP green fluorescence in C2C12 myotubes under fed and starved conditions (n = 1). (**b**) Experimental design for C2C12 myotube adenosine triphosphate reporter (iATP) assay; created with BioRender.com. MOI = multiplicity of infection; VEH = vehicle; PIP = pipecolic acid; SUC = succinate; PAS = pipecolic acid and succinate. Source data are provided in the Source Data file.
