# Supplementary material for: Exercise-associated microbial metabolites prevent skeletal muscle atrophy in adult female mice
Source: Nat Commun. 2026 Jul 10;17:8512. doi: 10.1038/s41467-026-74852-w (PMC13482395; doi:10.1038/s41467-026-74852-w)
Supplement: Supplementary file 2 [file 41467_2026_74852_MOESM2_ESM.pdf]

nature portfolio

Corresponding author(s): John J. McCarthy; Yuan Wen

Last updated by author(s): Apr 7, 2026

Reporting Summary

Nature Portfolio wishes to improve the reproducibility of the work that we publish. This form provides structure for consistency and transparency in reporting. For further information on Nature Portfolio policies, see our Editorial Policies and the Editorial Policy Checklist.

Statistics

For all statistical analyses, confirm that the following items are present in the figure legend, table legend, main text, or Methods section.

n/a	Confirmed	
☐	☒	The exact sample size (*n*) for each experimental group/condition, given as a discrete number and unit of measurement
☐	☒	A statement on whether measurements were taken from distinct samples or whether the same sample was measured repeatedly
☐	☒	The statistical test(s) used AND whether they are one- or two-sided<br>*Only common tests should be described solely by name; describe more complex techniques in the Methods section.*
☐	☒	A description of all covariates tested
☐	☒	A description of any assumptions or corrections, such as tests of normality and adjustment for multiple comparisons
☐	☒	A full description of the statistical parameters including central tendency (e.g. means) or other basic estimates (e.g. regression coefficient) AND variation (e.g. standard deviation) or associated estimates of uncertainty (e.g. confidence intervals)
☐	☒	For null hypothesis testing, the test statistic (e.g. *F*, *t*, *r*) with confidence intervals, effect sizes, degrees of freedom and *P* value noted<br>*Give P values as exact values whenever suitable.*
☒	☐	For Bayesian analysis, information on the choice of priors and Markov chain Monte Carlo settings
☒	☐	For hierarchical and complex designs, identification of the appropriate level for tests and full reporting of outcomes
☐	☒	Estimates of effect sizes (e.g. Cohen's *d*, Pearson's *r*), indicating how they were calculated

Our web collection on statistics for biologists contains articles on many of the points above.

Software and code

Policy information about availability of computer code

Data collection	ClockLab software (Actimetrics, Wilmette, IL) for running wheel data acquisition. Agilent MassHunter Quantitative Analysis v12 for targeted metabolomics. LabChart software (v8.1.19) for ex vivo muscle force recording. DMC v6.000 for in vivo torque data. Seahorse XF24 Analyzer software (Agilent) for mitochondrial complex activity. Olympus APEXVIEW APX100 software (Evident Scientific) for iATP fluorescence imaging. Cytation 5 Multimode reader software (Biotek) for citrate synthase assay. Agilent 2100 Bioanalyzer software for rRNA analysis.
Data analysis	GraphPad Prism v10.2.3 (GraphPad Software, La Jolla, CA) for all statistical analyses. MATLAB (ver. R2024b Update 5) for force/torque analyses. MetaPhlan 3.0 for metagenomic taxonomic classification. MicrobiomeAnalyst 2.0 and R v4.3.2 (packages: Vegan, DESeq2) for microbial diversity and differential abundance analyses. MetaboAnalyst 6.0 and MetaboAnalystR for metabolomics PLS-DA, random forest, and pathway analyses. Enrichr for gene ontology and pathway enrichment. R package DEP for proteomic data analysis; EnhancedVolcano for visualization. Compound Discoverer 3.3SP (ThermoFisher Scientific) for untargeted metabolomics mass spec analysis. ThermoFisher Scientific Proteome Discoverer v3.1 with SEQUEST algorithm for proteomics. MyoVision 2.0 for automated, blinded myofiber cross-sectional area analysis.

For manuscripts utilizing custom algorithms or software that are central to the research but not yet described in published literature, software must be made available to editors and reviewers. We strongly encourage code deposition in a community repository (e.g. GitHub). See the Nature Portfolio guidelines for submitting code & software for further information.

Data

Policy information about availability of data

All manuscripts must include a data availability statement. This statement should provide the following information, where applicable:

- Accession codes, unique identifiers, or web links for publicly available datasets
- A description of any restrictions on data availability
- For clinical datasets or third party data, please ensure that the statement adheres to our policy

All datasets generated during the course of this study will be made available upon reasonable request.

Research involving human participants, their data, or biological material

Policy information about studies with human participants or human data. See also policy information about sex, gender (identity/presentation), and sexual orientation and race, ethnicity and racism.

Reporting on sex and gender	This research did not involve human participants, their data, or biological material.
Reporting on race, ethnicity, or other socially relevant groupings	This research did not involve human participants, their data, or biological material.
Population characteristics	This research did not involve human participants, their data, or biological material.
Recruitment	This research did not involve human participants, their data, or biological material.
Ethics oversight	This research did not involve human participants, their data, or biological material.

Note that full information on the approval of the study protocol must also be provided in the manuscript.

Field-specific reporting

Please select the one below that is the best fit for your research. If you are not sure, read the appropriate sections before making your selection.

☒ Life sciences ☐ Behavioural & social sciences ☐ Ecological, evolutionary & environmental sciences

For a reference copy of the document with all sections, see nature.com/documents/nr-reporting-summary-flat.pdf

Life sciences study design

All studies must disclose on these points even when the disclosure is negative.

Sample size	No statistical methods were used to predetermine sample size. Sample sizes (n = 3-9 per group) were chosen based on our previous published work interrogating exercise training models and gut microbial regulation of skeletal muscle size, which demonstrated adequate power to detect meaningful differences in muscle phenotype. Specifically: cecal transfer experiments n = 9 per group; metabolite administration (atrophy) n = 5-6 per group; metabolite administration (exercise) n = 3 per group; in vitro experiments performed in triplicate.
Data exclusions	One REC-SED animal was excluded from metagenomic analysis due to insufficient sequencing read depth. One VEH sample was excluded from proteomic analysis as a statistical outlier identified during quality control. All other data were included in analyses.
Replication	The 10-day hindlimb immobilization model was validated in an independent dose-response cohort (Extended Data Fig. 5). Cecal transfer atrophy phenotype and PAS metabolite experiments were each performed in at 2-3 separate cohorts which were combined for final analyses. Ex vivo and in vivo muscle function assays were performed in additional independent cohorts. In vitro ATP reporter experiments were performed with biological replicates. All attempts at replication were successful.
Randomization	Animals were randomly assigned to experimental groups at study initiation. For cecal transfer experiments, sedentary donors were randomly assigned; exercised donors were pool-matched by total running distance to eliminate exercise volume as a confounding variable. For metabolite experiments, mice were randomly assigned to VEH, PIP, SUC, or PAS groups.
Blinding	Myofiber cross-sectional area quantification was performed using MyoVision 2.0, an automated software that analyzes images in a blinded manner. Full blinding to experimental group was not possible during tissue collection, exercise wheel monitoring, or gavage procedures because mice were housed according to treatment group. Western blot quantification and molecular analyses were performed without explicit blinding, though all data were analyzed using standardized automated pipelines where possible.

Reporting for specific materials, systems and methods

We require information from authors about some types of materials, experimental systems and methods used in many studies. Here, indicate whether each material, system or method listed is relevant to your study. If you are not sure if a list item applies to your research, read the appropriate section before selecting a response.

Materials & experimental systems

n/a	Involved in the study
☐	☒ Antibodies
☐	☒ Eukaryotic cell lines
☒	☐ Palaeontology and archaeology
☐	☒ Animals and other organisms
☒	☐ Clinical data
☒	☐ Dual use research of concern
☒	☐ Plants

Methods

n/a	Involved in the study
☒	☐ ChIP-seq
☒	☐ Flow cytometry
☒	☐ MRI-based neuroimaging

Antibodies

Antibodies used	Anti-myosin heavy chain Type I, Developmental Studies Hybridoma Bank (DSHB), cat no. BA.D5, Iowa City, IA; 1:100 dilution. Anti-myosin heavy chain Type IIA, DSHB, cat no. SC.71; 1:100. Anti-myosin heavy chain Type IIB, DSHB, cat no. BF.F3; 1:100. Rabbit anti-laminin IgG, Sigma-Aldrich, cat. no. L9393; 1:100. Anti-AKT, Cell Signaling Technology, cat. no. 9272S. Anti-phospho-AKT (Ser473), Cell Signaling Technology, cat. no. 4051S. Anti-p70S6K, Cell Signaling Technology, cat. no. 9202S. Anti-phospho-p70S6K (Thr389), Cell Signaling Technology, cat. no. 9206S. Anti-OXPHOS (mitochondrial complex proteins cocktail), ThermoFisher Scientific, cat. no. 45-8099.
Validation	BA.D5, SC.71, and BF.F3 are well-established, widely validated primary antibodies for murine skeletal muscle fiber typing used extensively in the field; validation citations are available from the DSHB and in numerous published studies. Anti-laminin (L9393, Sigma-Aldrich) is a validated antibody for labeling the muscle fiber basal lamina in mouse tissue. AKT, p-AKT, p70S6K, and p-p70S6K antibodies (Cell Signaling Technology) are validated for mouse skeletal muscle by the manufacturer and widely used in the muscle biology field. The OXPHOS cocktail (ThermoFisher 45-8099) is validated for detection of mitochondrial complexes I–V in mouse tissue by the manufacturer.

Eukaryotic cell lines

Policy information about cell lines and Sex and Gender in Research

Cell line source(s)	Mouse C2C12 myoblasts (ATCC, Manassas, VA; cat. no. CRL-1772). Sex: female (derived from female C3H mouse). HEK293T-17 cells were used for AAV production only (ATCC, cat. no. CRL-11268); sex: female.
Authentication	C2C12 and HEK293T-17 cell lines were obtained directly from ATCC and used within a low passage number. No additional authentication was performed beyond vendor certification.
Mycoplasma contamination	Cell lines were not explicitly tested for mycoplasma contamination during this study. Cultures were maintained under standard sterile conditions with regular microscopic inspection for contamination.
Commonly misidentified lines (See ICLAC register)	No commonly misidentified cell lines (per the ICLAC database) were used in this study.

Animals and other research organisms

Policy information about studies involving animals; ARRIVE guidelines recommended for reporting animal research, and Sex and Gender in Research

Laboratory animals	Female C57BL/6J mice (strain #000664), 4-12 months of age, were used for all experiments. Mice were obtained from The Jackson Laboratory and housed in the University of Kentucky vivarium with a 14h:10h light/dark cycle, controlled temperature (22 ± 1°C), and ad libitum access to standard chow and water. Mice were acclimated for ≥4 weeks prior to experimental procedures to allow gut microbiome stabilization.
Wild animals	This study did not involve wild animals.
Reporting on sex	All experiments were conducted exclusively in females. Sex was not a variable in the experimental design; findings are therefore reported for the female sex only. The use of female mice was chosen for consistency with our previously published exercise training and gut microbiome studies. Whether findings translate to male mice was not tested and remains to be determined.
Field-collected samples	This study did not involve samples collected from the field.
Ethics oversight	All animal procedures were approved by the Institutional Animal Care and Use Committee at the University of Kentucky and performed in accordance with the National Institutes of Health Guide for the Care and Use of Laboratory Animals.

Note that full information on the approval of the study protocol must also be provided in the manuscript.

Plants

Seed stocks	This research did not involve plants.
Novel plant genotypes	This research did not involve plants.
Authentication	This research did not involve plants.
