# Supplementary material for: Exercise-associated microbial metabolites prevent skeletal muscle atrophy in adult female mice
Source: Nat Commun. 2026 Jul 10;17:8512. doi: 10.1038/s41467-026-74852-w (PMC13482395; doi:10.1038/s41467-026-74852-w)
Supplement: Supplementary file 3 [file 41467_2026_74852_MOESM3_ESM.pdf]

nature portfolio

Peer Review File

Exercise-Associated Microbial Metabolites Prevent Skeletal Muscle Atrophy in Adult Female Mice

Corresponding Author: Dr John McCarthy

This file contains all reviewer reports in order by version, followed by all author rebuttals in order by version.

Version 0:

Reviewer comments:

Reviewer #1

(Remarks to the Author)
In this revised and transferred manuscript, Burke, Valentino and colleagues tested the hypothesis that microbiome-derived metabolites underpinned their prior observation that the gut microbiome plays a critical role in skeletal muscle adaptation to exercise. The authors transferred cecal effluent from exercise-trained or sedentary donors into exercise-naïve recipient mice undergoing unilateral hindlimb immobilization, observing that recipients of exercise-trained cecal effluent were resistant to immobilization-induced muscle atrophy. Using untargeted and targeted metabolomics and follow-up supplementation trials, they identified and confirmed two microbial metabolites — pipecolic acid and succinate — that could independently limit muscle atrophy and preserve muscle function in mice subjected to hindlimb immobilization. These experiments make a strong case for the development and testing of microbial metabolites as therapeutic agents in conditions of limited physical activity.

Crucially, the addition of metagenomic sequencing data addresses my previous major concern about the possible lack of transplant fidelity between donors and recipients. I agree with the authors that the lack of specific donor-recipient matching is not especially concerning given abundant evidence that treatment-induced profiles were at least reasonably well transferred to recipients.

However, a few (minor) concerns about the manuscript remain:

1. Were exercise levels measured in recipients post-transplant? If not, how can the authors disentangle the effects of exercise-induced changes in the donor microbiota toward a profile with higher pipecolic acid and succinate production versus potential microbiome-mediated transmissibility of voluntary exercise levels that stimulate higher in situ pipecolic acid and succinate production in recipients? In other words, is it possible that recipients of an exercised-conditioned microbiota experienced improved outcomes in part because they were motivated to exercise more despite unilateral hindlimb immobilization? This is an especially salient concern given the data presented in Figure 2M showing that recipients of pipecolic acid and succinate supplementation exhibited higher levels of voluntary wheel running than vehicle controls.

2. Among PAS-treated mice, we should expect succinate to be enriched in the metabolic analyses of muscle, similar to pipecolic acid. Was succinate also enriched, as expected? If not, can the authors speculate as to why not?

3. Lines 170-173: “Initially, succinic acid decreased in the cecal content (-76.2%, $p < 0.05$ by independent t-test), but was elevated by 24 hours (+4,129.7%, $p < 0.001$) along with non-significant reductions in the serum (-50.9%, $p = 0.13$) (Extended Data Fig. 6C-D).” What accounts for the supraphysiological rise in succinic acid 24 hours after PEG treatment?

4. Inocula consisted of pooled cecal contents from 3 sedentary or 3 exercised donors. The authors indicated that the 3 exercised donors were selected to match for exercise volume, which makes sense, but how were the 3 sedentary donors selected from among the 9 in that group?

5. Line 114: “after five weeks of cecal transfers” — although this information is provided in the Methods, it would help to briefly relay the schedule of cecal transfers in the main text so that readers can appreciate the level of intervention.

Reviewer #2

(Remarks to the Author)
The revised version has toned town the conclusion and acknowledged the limitation of this study.

Reviewer #3

(Remarks to the Author)
Burke and colleagues aimed at identifying microbiome-derived metabolites from exercised mice that confer protective, anti-atrophic effects in casting-immobilized mice. Cecal transfer, and the ensuing direct administration of two metabolites, was successful in mitigating soleus, but not plantaris fiber cross-sectional area. The authors could demonstrate functional consequence of this rescue by determining ex vivo and in vivo contractile force. Moreover, they demonstrated association with ribosomal proteins and rRNA, leading to the speculation that these metabolites could affect muscle proteostasis. Overall, the presented data are interesting, and the proposed link between microbiome-derived metabolites and training adaptation intriguing. Nevertheless, the following points might be considered:

1.) An obvious disconnect between the changes in muscle mass (for both soleus and plantaris), and the alterations in soleus fiber size cross-sectional area exists, in the cecal transfer as well as in the PAS treatment. How do the authors reconcile this? Is fiber number changed? Why are no data for type IIX fibers reported (neither for soleus nor plantaris)? Presumably, the reduction in soleus type IIA fibers in the REC-SED is offset by a relative shift towards a higher proportion of type IIX fibers?

2.) The choice of muscle seems somewhat arbitrary. In the cecal transfer, the only effect was seen in soleus, while the plantaris remained unaffected. Moreover, the rescue in cross-sectional area was limited to type I fibers in the soleus. Based on these findings, the functional assessment of soleus seems reasonable. However, in the PAS treated mice, a rescue of both type I and type IIA fiber cross-sectional area was found. Second, an improvement in torque production in plantar flexion was reported, which is mainly mediated by soleus, plantaris, and mostly (!) gastrocnemius. It thus is puzzling why no other muscle besides soleus was analyzed: maybe the effect of PA extends even to type IIX and IIB fibers? Or, alternatively, would also be expected at least in gastrocnemius, which harbors a significant number of type IIA fibers. Thus, muscle weights and cross-sectional areas of all three muscles should be shown, in particular the gastrocnemius for which proteomics was done. Otherwise, the link between the morphological/functional assessment (almost exclusively soleus), and the mechanistic/proteomic study (gastrocnemius) remains tenuous.

3.) Some of the conclusions should be toned down: for example, even though no significant change in respiration and OXPHOS protein levels were found in vivo, the authors speculate that “Instead, our work demonstrates promising effects of PAS treatment on ATP levels and mitochondrial complex activity.”, which is only based on increased citrate synthase activity in treated C2C12 cells.

Version 1:

Reviewer comments:

Reviewer #3

(Remarks to the Author)
The authors have addressed all of my points in an adequate manner.

Open Access This Peer Review File is licensed under a Creative Commons Attribution 4.0 International License, which permits use, sharing, adaptation, distribution and reproduction in any medium or format, as long as you give appropriate credit to the original author(s) and the source, provide a link to the Creative Commons license, and indicate if changes were made.
In cases where reviewers are anonymous, credit should be given to 'Anonymous Referee' and the source.
The images or other third party material in this Peer Review File are included in the article's Creative Commons license, unless indicated otherwise in a credit line to the material. If material is not included in the article's Creative Commons license and your intended use is not permitted by statutory regulation or exceeds the permitted use, you will need to obtain permission directly from the copyright holder.
To view a copy of this license, visit https://creativecommons.org/licenses/by/4.0/

Response to Final Nature Communications Reviewer's Comments

Thank you for accepting our manuscript, we are happy to have our findings distributed to the *Nature Communications* community. Below we detail the revisions we made in response to the final comments by reviewers.

Reviewer #1 Remarks to the Author:

1. "In this revised and transferred manuscript, Burke, Valentino and colleagues tested the hypothesis that microbiome-derived metabolites underpinned their prior observation that the gut microbiome plays a critical role in skeletal muscle adaptation to exercise. The authors transferred cecal effluent from exercise-trained or sedentary donors into exercise-naïve recipient mice undergoing unilateral hindlimb immobilization, observing that recipients of exercise-trained cecal effluent were resistant to immobilization-induced muscle atrophy. Using untargeted and targeted metabolomics and follow-up supplementation trials, they identified and confirmed two microbial metabolites — pipecolic acid and succinate — that could independently limit muscle atrophy and preserve muscle function in mice subjected to hindlimb immobilization. These experiments make a strong case for the development and testing of microbial metabolites as therapeutic agents in conditions of limited physical activity. Crucially, the addition of metagenomic sequencing data addresses my previous major concern about the possible lack of transplant fidelity between donors and recipients. I agree with the authors that the lack of specific donor-recipient matching is not especially concerning given abundant evidence that treatment-induced profiles were at least reasonably well transferred to recipients."
    a. **Response:** We thank the reviewer for their balanced review which acknowledges the strengths of the present manuscript. We have revised based on the critiques below to further clarify our findings.
2. "However, a few (minor) concerns about the manuscript remain: Were exercise levels measured in recipients post-transplant? If not, how can the authors disentangle the effects of exercise-induced changes in the donor microbiota toward a profile with higher pipecolic acid and succinate production versus potential microbiome-mediated transmissibility of voluntary exercise levels that stimulate higher in situ pipecolic acid and succinate production in recipients? In other words, is it possible that recipients of an exercised-conditioned microbiota experienced improved outcomes in part because they were motivated to exercise more despite unilateral hindlimb immobilization? This is an especially salient concern given the data presented in Figure 2M showing that recipients of pipecolic acid and succinate supplementation exhibited higher levels of voluntary wheel running than vehicle controls."
    a. **Response:** The recipient mice did not perform any exercise during the course of CMT experiments. After their final CMT, they were immobilized for 10 days, then euthanized for tissue collection. Therefore, we conclude that any effects observed were exclusively due to the treatment (i.e., cecal transfers). However, to the reviewer's point, we did not measure any daily activity of the recipients during immobilization. While the casting renders normal cage ambulation

challenging, anecdotally, during our daily health check, we did not notice any marked difference in the activity levels of the two groups during the period of hindlimb immobilization. The fiber cross-sectional area (CSA) of the non-casted legs also provides some insight into this consideration. If the CMTs from exercised donors resulted in increased activity such that muscle atrophy was mitigated, it is reasonable to expect that the contralateral control leg might be affected by the increased activity as well. Since there were no differences in fiber CSA between the control legs of each group (Fig. 1E, Fig. 2B), it seems likely that any differences in activity between recipient groups had minimal to no effect on muscle size.

   b. We have edited the manuscript to make special note of the absence of exercise in recipient mice:
      i. Lines 26-28: "To identify such exercise-associated microbial metabolites, we transferred cecal contents from exercise-trained donor mice into exercise-naïve recipient mice undergoing unilateral hindlimb immobilization."
      ii. Lines 50-54: "To test our hypothesis, we first transferred cecal content from sedentary donors (DON-SED) or exercise-trained donors (DON-EXR) into exercise-naïve recipient mice (REC-SED and REC-EXR, respectively) that were undergoing muscle atrophy induced by unilateral hindlimb immobilization (HLI)."
      iii. Lines 108-109: "After seven cecal transfers over the course of five weeks, we induced muscle atrophy in exercise-naïve recipient mice through 10 days of unilateral HLI via casting…"

3. "Among PAS-treated mice, we should expect succinate to be enriched in the metabolic analyses of muscle, similar to pipecolic acid. Was succinate also enriched, as expected? If not, can the authors speculate as to why not?"
   a. **Response:** Succinate was not enriched in muscle with PAS treatment. Succinate is an important intermediate metabolite which is promptly metabolized in multiple fundamental metabolic pathways (i.e., TCA cycle, electron transport chain). We detected slight, non-significant elevation in several derivatives and succinylated species in muscle tissue. Therefore, it is possible that the exogenous succinate was shuttled through various pathways. However, this would require confirmation via stable isotope tracing. Since we do not have these data, we omit this speculation from the manuscript.
4. "Lines 170-173: 'Initially, succinic acid decreased in the cecal content (-76.2%, $p < 0.05$ by independent t-test), but was elevated by 24 hours (+4,129.7%, $p < 0.001$) along with non-significant reductions in the serum (-50.9%, $p = 0.13$) (Extended Data Fig. 6C-D).' What accounts for the supraphysiological rise in succinic acid 24 hours after PEG treatment?"
   a. **Response:** The PEG treatment resulted in a disruption of the gut microbiome, which demonstrated the sensitivity of both pipecolic acid and succinate to gut microbiome perturbations. The increase in succinate 24-hours post-treatment likely reflects a disruption of the ratio of succinate-producing to succinate-

consuming microbes in the absence of subsequent colonization (e.g., cecal microbial transfers). However, we did not perform metagenomic sequencing on PEG-treated mice and therefore limit the conclusions we draw from these data to those mentioned above.

5. "Inocula consisted of pooled cecal contents from 3 sedentary or 3 exercised donors. The authors indicated that the 3 exercised donors were selected to match for exercise volume, which makes sense, but how were the 3 sedentary donors selected from among the 9 in that group?"
    a. **Response:** Thank you for the requested clarification. The sedentary donors were randomly assigned. We have edited the manuscript to include this information:
        i. Lines 418-421: "Cecal contents collected from nine exercise-trained or sedentary mice were pooled in groups of three (randomly assigned for sedentary donors, matched by running volume for exercised donors; Fig. 1B) and transferred via oral gavage (200μL per transfer) to anesthetized recipient mice six hours after the final PEG treatment."
6. "Line 114: "after five weeks of cecal transfers" — although this information is provided in the Methods, it would help to briefly relay the schedule of cecal transfers in the main text so that readers can appreciate the level of intervention."
    a. **Response:** We have revised the manuscript to clarify the timeline:
        i. Lines 108: "After seven cecal transfers over the course of five weeks…"
        ii. Lines 421-423: "Recipient mice received transfers on three consecutive days, followed by a single transfer per week for the next four weeks, resulting in seven total transfers over the course of five weeks (Fig. 1A)."

Reviewer #2 Remarks to the Author:

1. "The revised version has toned town the conclusion and acknowledged the limitation of this study."
    a. **Response:** Thank you for the positive evaluation and acceptance of our manuscript.

Reviewer #3 Remarks to the Author:

1. "Burke and colleagues aimed at identifying microbiome-derived metabolites from exercised mice that confer protective, anti-atrophic effects in casting-immobilized mice. Cecal transfer, and the ensuing direct administration of two metabolites, was successful in mitigating soleus, but not plantaris fiber cross-sectional area. The authors could demonstrate functional consequence of this rescue by determining ex vivo and in vivo contractile force. Moreover, they demonstrated association with ribosomal proteins and rRNA, leading to the speculation that these metabolites could affect muscle proteostasis. Overall, the presented data are interesting, and the proposed link between microbiome derived metabolites and training adaptation intriguing."
    a. **Response:** We thank the reviewer for highlighting the novelty of our findings and providing critiques to strengthen the paper. We outline our revisions below.
2. "Nevertheless, the following points might be considered: An obvious disconnect between the changes in muscle mass (for both soleus and plantaris), and the alterations in soleus

fiber size cross-sectional area exists, in the cecal transfer as well as in the PAS treatment. How do the authors reconcile this? Is fiber number changed? Why are no data for type IIX fibers reported (neither for soleus nor plantaris)? Presumably, the reduction in soleus type IIA fibers in the REC-SED is offset by a relative shift towards a higher proportion of type IIX fibers?"

   a. **Response:** We found no evidence of fiber number change in the soleus or plantaris. The discrepancy between mass and cross-sectional area (CSA) is likely due to such confounding factors as the precision of muscle excision, edema, and glycogen levels. We therefore provide CSA as the gold standard quantification of myofiber size. For the soleus, type IIx fibers are present in very low proportions which can preclude reliable quantification. As for the plantaris, hybrid fiber population renders unambiguous determination of type IIb versus type IIx fibers challenging. Therefore, in order to report sufficient and consistent fiber-type representation across all groups to allow meaningful statistical comparison, we limited our analysis to type I and type IIa in the soleus and type IIa and type IIb/x in the plantaris (Fig. 1G-L, Fig. 2C-F, Extended Data Fig. 7C-F).
   b. We have revised the manuscript to explain possible discrepancies between muscle mass and CSA:
      i. Lines 119-124: "Soleus and plantaris (a slow- and fast-twitch muscle, respectively) mass were decreased in both REC-SED and REC-EXR groups following HLI ($p < 0.0001$) (Fig. 1C-D). The gross measurement of muscle mass may be confounded by various factors such as the precision of muscle excision, edema, and glycogen levels, which can obscure true changes in myofiber size. We therefore assessed muscle size via CSA, the gold standard for directly quantifying myofiber size."

3. "The choice of muscle seems somewhat arbitrary. In the cecal transfer, the only effect was seen in soleus, while the plantaris remained unaffected. Moreover, the rescue in cross-sectional area was limited to type I fibers in the soleus. Based on these findings, the functional assessment of soleus seems reasonable. However, in the PAS treated mice, a rescue of both type I and type IIA fiber cross-sectional area was found. Second, an improvement in torque production in plantar flexion was reported, which is mainly mediated by soleus, plantaris, and mostly (!) gastrocnemius. It thus is puzzling why no other muscle besides soleus was analyzed: maybe the effect of PA extends even to type IIX and IIB fibers? Or, alternatively, would also be expected at least in gastrocnemius, which harbors a significant number of type IIA fibers. Thus, muscle weights and cross-sectional areas of all three muscles should be shown, in particular the gastrocnemius for which proteomics was done. Otherwise, the link between the morphological/functional assessment (almost exclusively soleus), and the mechanistic/proteomic study (gastrocnemius) remains tenuous."
   a. **Response:** Identical morphological assessments (i.e., mass, mean fiber CSA, fiber-type specific CSA, fiber-type distribution) are shown for slow-twitch (i.e., soleus) and fast-twitch (i.e., plantaris) muscles for both the cecal transfers (Fig. 1C-L) and metabolite treatment experiments (Fig. 2A-F, Extended Data Fig. 7A-F), which include fiber-type-specific data on type I, type IIa, and type IIb/x fibers.

Both CMTs and PAS are shown to effect muscle size in both muscle types to varying degrees (Fig. 1G-H, Fig. 2B, Extended Data. Fig. 7B). Immunohistochemistry of the gastrocnemius was not included since 1) a larger muscle was necessary for metabolomics and 2) a fast-twitch muscle (i.e., plantaris) was already assessed. *Ex vivo* muscle function was shown in the soleus (Fig. 1M-O, Fig 2. H-J), the murine muscle most comparable to human muscle in terms of fiber-type composition and transcriptome. *In vivo* muscle function captures the function of all plantarflexors, demonstrating the positive impact of PAS on the gastrocnemius, plantaris, and soleus as a group (Fig. 2K). Collectively, these data allow us to reasonably conclude that PAS has positive effects on muscle size and strength in both slow- and fast-twitch muscles.

b. We have revised the manuscript to highlight the impact of treatments, specifically PAS, on both slow- and fast-twitch muscles and further explained our rationale for focusing specific effort on the soleus:
    i. Lines 138-141: "To assess whether the reduction in skeletal muscle atrophy affected muscle function, we performed ex vivo muscle function analyses of the soleus, the murine muscle most representative of human muscle in terms of fiber-type composition and transcriptome[11], following HLI."
    ii. Lines 179-183: "Soleus muscle mass decreased in every group ($p < 0.01$ to 0.0001) (Fig. 2A); however, soleus mean muscle fiber CSA was maintained by PAS treatment (Fig. 2B) unlike vehicle (VEH) ($p < 0.01$), PIP ($p < 0.01$), and SUC groups ($p < 0.05$). We observed similar results in the plantaris muscle (Extended Data Fig. 7A-F)."
    iii. Lines 194-200: "PAS treatment preserved in vivo torque production at 80-200Hz ($p < 0.05$; area under curve $p < 0.0001$) (Fig. 2K), as well as maximal torque production ($p < 0.05$) (Fig. 2L), indicating the preservation of the function of entire muscle groups (i.e., plantar flexors) consisting of slow- and fast-twitch muscles in their native environments. Collectively, these results demonstrate that microbial metabolites associated with an exercise-trained gut microbiome can prevent skeletal muscle atrophy and preserve muscle function in slow- and fast-twitch muscles when directly administered to mice undergoing HLI."

4. "Some of the conclusions should be toned down: for example, even though no significant change in respiration and OXPHOS protein levels were found in vivo, the authors speculate that "Instead, our work demonstrates promising effects of PAS treatment on ATP levels and mitochondrial complex activity.", which is only based on increased citrate synthase activity in treated C2C12 cells."
    a. **Response:** We conclude that PAS treatment impacts ATP levels and mitochondrial complex activity based on 1) an increase in ATP levels both *in vivo* (Fig. 3I) and *in vitro* (Fig. 3N) with PAS treatment, 2) trending increases in *in vivo* complex activity (Fig. 3K-L), and 3) increased CS activity *in vitro* (Fig. 3O). Given these multiple lines of evidence, we are confident with our assertions regarding the impact of PAS on cellular respiration.

b. These results are highlighted below:
    i. Lines 254-261: "Along with higher pipecolic acid levels ($p < 0.0001$) (Fig. 3H), we observed significantly higher levels of ATP with PAS treatment ($p < 0.05$) (Fig. 3I), suggesting increased energy availability. Moreover, hypoxanthine, an adenine nucleotide degradation product, was lower in PAS-treated muscle ($p < 0.01$) (Fig. 3J). This is noteworthy as increased adenine nucleotide degradation is associated with skeletal muscle atrophy[18]. We assessed mitochondrial respiration, finding that the activity of mitochondrial complexes II ($p = 0.07$) and IV ($p = 0.09$) (Fig. 3K-L) were trending higher in the PAS group, despite no change in the expression of any of the 5 OXPHOS complexes (Extended Data Fig. 10A-E)."
    ii. Lines 265-269: "Next, we treated iATP+ myotubes with VEH, PIP, SUC, or PAS for three days (Extended Data Fig. 11B) and quantified fluorescence to determine ATP levels. PAS treatment increased ATP levels more than VEH, SUC or PIP ($p < 0.0001$) (Fig. 3M-N). The elevation in ATP level was accompanied by higher citrate synthase activity in PIP ($p < 0.01$) and PAS ($p < 0.001$) relative to VEH (Fig. 3O)."
